# Thymic Microenvironment Is Modified by Malnutrition and *Leishmania infantum* Infection

**DOI:** 10.3389/fcimb.2019.00252

**Published:** 2019-07-12

**Authors:** Monica Losada-Barragán, Adriana Umaña-Pérez, Jonathan Durães, Sergio Cuervo-Escobar, Andrés Rodríguez-Vega, Flávia L. Ribeiro-Gomes, Luiz R. Berbert, Fernanda Morgado, Renato Porrozzi, Daniella Arêas Mendes-da-Cruz, Priscila Aquino, Paulo C. Carvalho, Wilson Savino, Myriam Sánchez-Gómez, Gabriel Padrón, Patricia Cuervo

**Affiliations:** ^1^Laboratório de Pesquisa em Leishmanioses, Instituto Oswaldo Cruz, Fiocruz, Rio de Janeiro, Brazil; ^2^Grupo de Investigación en Biología Celular y Funcional e Ingeniería de Biomoléculas, Departamento de Biologia, Universidad Antonio Nariño, Bogotá, Colombia; ^3^Grupo de Investigación en Hormonas, Departamento de Química, Universidad Nacional de Colombia, Bogotá, Colombia; ^4^Facultad de Ciencias, Universidad de Ciencias Aplicadas y Ambientales, Bogotá, Colombia; ^5^Laboratório de Pesquisa em Malária, Instituto Oswaldo Cruz, Fiocruz, Rio de Janeiro, Brazil; ^6^Laboratório de Pesquisas sobre o Timo, Instituto Oswaldo Cruz, Fiocruz, Rio de Janeiro, Brazil; ^7^Instituto Nacional de Ciência e Tecnologia em Neuroimunomodulação, Fiocruz, Rio de Janeiro, Brazil; ^8^Instituto Leônidas e Maria Deane, Fiocruz, Manaus, Brazil; ^9^Computational Mass Spectrometry and Proteomics Group, Fiocruz, Rio de Janeiro, Brazil

**Keywords:** thymus, thymic microenvironment, protein malnutrition, *Leishmania infantum*, visceral leishmaniasis, interstitial fluid, proteomics, fatty acid oxidation

## Abstract

Detrimental effects of malnutrition on immune responses to pathogens have long been recognized and it is considered a main risk factor for various infectious diseases, including visceral leishmaniasis (VL). Thymus is a target of both malnutrition and infection, but its role in the immune response to *Leishmania infantum* in malnourished individuals is barely studied. Because we previously observed thymic atrophy and significant reduction in cellularity and chemokine levels in malnourished mice infected with *L. infantum*, we postulated that the thymic microenvironment is severely compromised in those animals. To test this, we analyzed the microarchitecture of the organ and measured the protein abundance in its interstitial space in malnourished BALB/c mice infected or not with *L. infantum*. Malnourished-infected animals exhibited a significant reduction of the thymic cortex:medulla ratio and altered abundance of proteins secreted in the thymic interstitial fluid. Eighty-one percent of identified proteins are secreted by exosomes and malnourished-infected mice showed significant decrease in exosomal proteins, suggesting that exosomal carrier system, and therefore intrathymic communication, is dysregulated in those animals. Malnourished-infected mice also exhibited a significant increase in the abundance of proteins involved in lipid metabolism and tricarboxylic acid cycle, suggestive of a non-proliferative microenvironment. Accordingly, flow cytometry analysis revealed decreased proliferation of single positive and double positive T cells in those animals. Together, the reduced cortical area, decreased proliferation, and altered protein abundance suggest a dysfunctional thymic microenvironment where T cell migration, proliferation, and maturation are compromised, contributing for the thymic atrophy observed in malnourished animals. All these alterations could affect the control of the local and systemic infection, resulting in an impaired response to *L. infantum* infection.

## Introduction

The thymus gland is the central lymphoid organ in the adaptive immune system, where maturation, proliferation, and exportation of T lymphocytes take place. The successful development of mature T cells depends on the constant migration of differentiating thymocytes through the thymic microenvironment. The mechanisms directing this migration are heavily dependent on the thymic microenvironment, which regulates the process of T cell development through surface molecules, extracellular matrix (ECM) proteins, matrix metallopeptidases, and by secreting soluble polypeptides as cytokines, chemokines, and hormones (Smythe et al., 1971; Schonland, 1972; Aref et al., 1982; Jambon et al., 1988; Aaby et al., 2002; Gameiro et al., 2010). However, the global complexity of soluble protein components that mediate T cell development in this environment is to be better defined, both in physiological and pathological conditions.

It is widely recognized that thymus is one of the most affected organs during malnutrition. In malnourished children, histological analyses revealed thymus atrophy (Schonland, 1972), with thymocyte depletion, increased intra and inter-lobular connective tissue, and decreased cortico-medullary limits (Smythe et al., 1971; Aref et al., 1982; Jambon et al., 1988). Importantly, it has been shown that a smaller thymus is a consistent and independent risk factor for mortality and is predictive of immune competence (Aaby et al., 2002; Garly et al., 2008).

Interestingly, several infectious diseases induce thymic atrophy and altered T cell subsets (Mendes-Da-Cruz et al., 2003; Savino et al., 2007; Andrade et al., 2008; Liu et al., 2014), thus sharing these characteristics with the effects of malnutrition in the thymus. In experimental models, the detrimental effects of malnutrition and infection on immunity have long been recognized (Savino and Dardenne, 2010; Ibrahim et al., 2013, 2017; Akuffo et al., 2018). Nevertheless, few studies evaluated the coexistence of infection and malnutrition (Savino, 2006; Perez et al., 2012; Cuervo-Escobar et al., 2014; Losada-Barragan et al., 2017; Zacarias et al., 2017). Acute infection by *Trypanosoma cruzi* leads to thymic atrophy and thymocyte depletion mainly in the cortical region of the organ (Savino and Dardenne, 2010), and this change is further exacerbated during protein malnutrition (Akingbemi et al., 1996).

We previously demonstrated that the thymus of protein malnourished mice infected with *L. infantum* presents severe atrophy and reduced absolute cellularity, including a drastic decrease of CD4^+^CD8^+^ thymocytes and significant decrease in single positive T cell total numbers (Cuervo-Escobar et al., 2014). In addition, parasites were detected in the thymus of both well-nourished and malnourished animals infected with *L. infantum* but amastigote nests were only observed in malnourished mice (Losada-Barragan et al., 2017). We also observed that protein malnutrition does affect thymocyte migration of *L. infantum*-infected animals, rather than increasing their apoptosis. In fact, protein malnutrition induced a significant reduction of thymic contents of chemokines such as CCL5, CXCL12, CXCL9, and CXCL10 as well as IGF1 in infected animals, suggesting altered migratory capabilities of developing T cells. However, since in this combined condition developing thymocytes were able to migrate *ex vivo* in response to chemotactic stimuli, our data indicated that malnutrition may compromise the thymic microenvironment rather than migratory capability of T cells *per se* (Losada-Barragan et al., 2017). Those results, together with the observation of an early increase in the splenic parasite load in malnourished animals, suggested that a precondition of malnutrition is affecting cell-mediated immune response to *L. infantum* (probably by altering local and systemic T cell migration) leading to an altered capacity of protein-deprived animals to control parasite spreading and proliferation (Losada-Barragan et al., 2017). In this work, we hypothesized that protein malnutrition alters thymic microarchitecture and abundance of intrathymic soluble proteins that mediate cell-matrix and cell-cytokine/chemokine mediated interaction leading to defects upon T cell maturation and migration, resulting in a severe clinical outcome during *L. infantum* infection. Accordingly, we conducted a quantitative mass spectrometry-based proteomics analysis of the interstitial fluid of the thymus in protein malnourished mice infected with *L. infantum* accompanied by a histopathological study of the thymic microarchitecture. This first descriptive work of the thymic microenvironment in the light of proteomics revealed significant intrathymic alterations related to T cell migration, maturation, and proliferation that could contribute to a defective immune response during visceral leishmaniasis.

## Materials and Methods

### Ethics Statement

This study was carried out in accordance with the recommendations of the Guide for the Care and Use of Laboratory Animals of the National Institutes of Health—Eighth Edition. The protocol was approved by the Instituto Oswaldo Cruz committee for Animal Care and Use (License #LW-27/14). The *L. infantum* strain MCAN/BR/2000/CNV-FEROZ used in this study was provided by the Collection of *Leishmania* of the Instituto Oswaldo Cruz, Rio de Janeiro (Coleção de *Leishmania* do Instituto Oswaldo Cruz, CLIOC; http://clioc.fiocruz.br/). This collection is registered in the World Federation for Culture Collections (WFCC-WDCM 731) and is recognized as a Depository Authority by the Brazilian Ministry of the Environment (D.O.U. 05.04.2005).

### Parasite Culture

Parasites were cultivated at 25°C in Schneider's medium containing 10% fetal calf serum (FCS) and were collected at the stationary phase by centrifugation at 1,800 *g* for 5 min. The parasites were then washed twice in PBS, pH 7.2.

### Mice, Feeding Protocol, and Experimental Infection

BALB/c AnUnib (male, 3 weeks old) mice were purchased from Centro Multidisciplinar para Investigação Biológica na Área da Ciência em Animais de Laboratório da Universidade Estadual de Campinas (CEMIB-UNICAMP, http://www.cemib.unicamp.br/servicos/cadastro_linhagens.php). Mice were kept in ventilated cages, in a 12/12 h light/dark cycle, in a specific pathogen–free area of the mouse facility of the Instituto Oswaldo Cruz (IOC-FIOCRUZ). Experimental infection was conducted as previously described (Cuervo-Escobar et al., 2014; Losada-Barragan et al., 2017). Animals were provided with a diet containing 14% protein (MP Biomedicals, Inc., USA, Catalog No. 960258). After 1 week of acclimation, animals were randomly divided into two groups: one group being fed 14% (13,79 g crude protein per 100 g food pellets; control protein, CP) and another group being fed a 4% protein content diet (4,59 g crude protein per 100 g food pellets; low protein, LP) (MP Biomedicals, Inc., USA, Catalog No 960254). These two diets are isocaloric; each providing 3.7 kcal/g. The caloric protein deficiency in the 4% protein diet was replaced by additional carbohydrate calories. The animals had free access to water and food. Food rations per cage were daily weighed and feed consumption was calculated (Sanchez-Gomez et al., 1999). After 7 days of diet, each group was divided into two subgroups, and one subgroup of each diet group was infected intravenously (tail vein) with 1 × 10^7^ parasites, whereas the other group received saline solution. Diets were maintained after infection. Body weight was recorded every third day, but mice were monitored daily during the course of infection (14 days). The animals were euthanized after 14 days post-infection; euthanasia was conducted according to the protocol approved by license LW-27/14. Briefly, animals were anesthetized intraperitoneally with a mix of 10 mg/kg xylazine−200 mg/kg ketamine. When anesthetized, the animals were exposed to carbon dioxide gas. The thymus was removed, weighed, and subsequently processed for interstitial fluid (IF) extraction. Relative and absolute cell numbers in thymus were estimated by hemocytometer counting.

### Flow Cytometry Analysis and Gating Strategies

The cells collected from the thymus were analyzed by flow cytometry according to previously described protocols (Cuervo-Escobar et al., 2014). Briefly, one million cells (1 × 10^6^) were incubated with an anti-Fc-γ III/II (CD16/32) receptor Ab (2.4G2, BD Biosciences) in PBS containing 2% FCS, followed by surface staining with fluorochrome-conjugated antibodies for 20 min at 4°C. The following anti-mouse antibodies were used: APC anti-CD3 (17A2), PE anti-CD4 (GK1.5), APC-Cy7 anti-CD8a (53-6.7), or with IgG isotype-matched control antibodies. To analyze the percentage of proliferative cells, CD4^+^, CD8^+^, and CD4^+^CD8^+^ (DP) T cells were treated with the Foxp3 Fixation/Permeabilization Buffer (eBioscience) according to the manufacturer's instructions, and then stained with FITC anti-Ki67 (16A8) for 30 min at room temperature. All antibodies were from BioLegend. Acquisition (100,000 events) was performed in a FACSCanto™ flow cytometer. On-line analysis was performed with FlowJo™ Software, version 8.7. To identify the population of interest we performed the following gate strategy: by first gating on alive (SSC vs. FSC) and singlets (FSC-H vs. FSC-A) cells, we defined the CD4 T cells as CD4^+^CD8^−^, CD8 T cells as CD4^−^CD8^+^, and double positive (DP) T cells as CD4^+^CD8^+^ T cells. A Fluorescence Minus One (FMO) Control was used to identify and gate the Ki67^+^ cells.

### Isolation of Interstitial Fluid From the Thymus

Interstitial fluid (IF) was obtained by injection of PBS and gentle wash of the thymus with sterile needle syringes. Intact cells were pelleted by centrifugation and supernatants were recovered for proteomic analysis, these supernatants are considered as enriched IF from the thymus. The collected fluids were concentrated to a final volume of 100 μL using Amicon Centrifugal Filters 3K (Millipore). To each fluid sample was added 0.1% of Rapigest (Waters) for protein denaturation and rupture of exosomes membrane.

### ITRAQ Labeling

**Figure 1:**
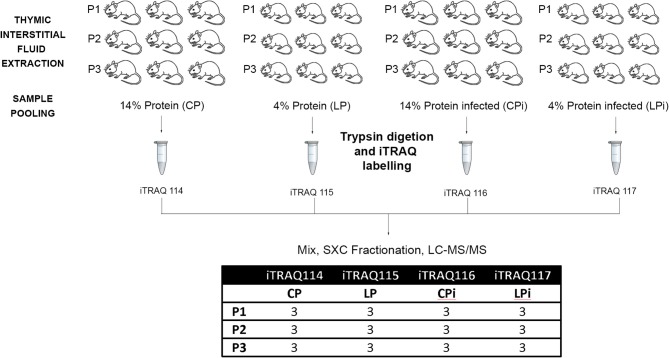
Workflow of the proteomics procedure for analyzing thymic interstitial fluid in protein malnourished and infected mice. Experimental workflow for the mass spectrometry-based identification and quantification of the IF proteins.

In order to adjust the study within a single multiplex experiment, pools of samples from different mice were used instead of individual samples. Equal amounts of fluid samples from three animals within each experimental condition were mixed to produce one sample pool (Figure 1). Three sample pools of each group (P1, P2, P3) were used for a total of nine different animals in each experimental condition. Therefore, three biological replicates and two technical replicates for each treatment were incorporated into the multiplex iTRAQ design.

Pooled samples were digested with lysyl endopeptidase mass spectrometry grade (Wako) and sequencing grade modified trypsin (Promega, Madison, WI, USA). After digestion, Rapigest was precipitated and peptides were desalted using Spin Column C18 (Harvard Apparatus, USA) according to the manufacturer's protocol. Peptides were labeled with iTRAQ 4-plex (Reagents iTRAQ Applications 4-plex Kit; AB Sciex, Framingham, MA, USA) and mixed as follows: iTRAQ114 for sample pools of animals fed 14% protein diet); iTRAQ115 for sample pools of animals fed 4% protein diet; iTRAQ116 for sample pools of animals fed 14% protein diet and infected; and iTRAQ117 for sample pools of animals fed 4% protein diet and infected. Labeled peptides were further mixed (Figure 1), fractionated by strong cation exchange using Macro Spin Columns (Harvard Apparatus, USA) and desalted using Spin Column C18 (Harvard Apparatus, USA).

### Mass Spectrometry Analysis

Tagged and desalinated fractions were subjected to nano-liquid chromatography coupled to mass spectrometry in tandem (nLC-MS/MS) in the mass spectrometry facility of the Carlos Chagas Institute—Fiocruz-Paraná. The nano-liquid chromatography system used was the *Easy NLC-1000* (*Thermo Fisher Scientific*). Injections were made in duplicate. In this system, peptides were loaded onto a reverse phase column packed in house with a flow of 500 nL/min with stationary phase ReprosilPur C18 Acqua (beads 1.9 m in diameter, Dr. Maisch) with a length of 30 cm and inner diameter of 75 μm. The elution of these peptides occurred with a flow of 250 nL/min applying a chromatographic gradient of 5–40% of phase B (5% DMSO, 0.1% formic acid in acetonitrile) for 180 min. Phase A was 5% DMSO in 0.1% formic acid.

Spectra were acquired in a LTQ Orbitrap XL-ETD mass spectrometer (Thermo Fisher Scientific) by data dependent acquisition (DDA), automatically switching between full scan MS (m/z 300-2000) at resolution of 60,000 (m/z 100) and MS/MS with dynamic exclusion of 90s. The five most intense ions with +2 and +3 charges were isolated and fragmented by higher energy collision dissociation (HCD) using normalized collision energy of 45 and 30 ms of activation time. All scan functions of the mass spectrometer and the gradients in the nLC were controlled by Xcalibur 2.0 software (*Thermo Fisher Scientific*).

### Protein Identification and Quantification

Protein identification was performed with PatternLab for Proteomics version 3.2.0.3 (http://patternlabforproteomics.org/) using the Comet PSM as search engine (Eng et al., 2013). *Mus musculus* protein sequences were download from UniProt database in February 2015 (http://www.uniprot.org/) with 83.653 entries. For estimation of false discovery rate (FDR), we subsequently generated a target-decoy database including the sequences of common mass spectra contaminants using the PatternLab's Search Database Generator (Carvalho et al., 2012). A cutoff score was established for accept a FDR of 1% based on the marked decoys from the database. The search parameters were tryptic and semi-tryptic peptide candidates, three missed cleavages, fixed modification of cysteine carbamidomethylation, fixed iTRAQ (+144.1 Da) at the N-terminal and in lysine (K) side chain with a 40 ppm mass tolerance of the precursor. Validation of the peptide-spectrum matches was done using Search Engine Processor (SEPro) integrated in the PatternLab for proteomics (Carvalho et al., 2012). The results were processed to accept only sequences with <5 ppm and two or more independent evidence of the presence of the protein in the sample (e.g., identifying a peptide with a different charge state, the same modified version of the unmodified peptide, or two different peptides for the same protein).

Relative quantification was performed using PatternLab's “Isobaric Analyzer” module. The SEPro file was used to produce a report of quantified peptides (Carvalho et al., 2009) A paired comparison analysis between the CP group and each of the other groups (LP, CPi, or LPi) was made, accepting at least 2 unique peptides per protein, 0.30 of “*peptide Log Fold Change Cutoff* ” and 0.05 of “*Peptide p-value Cutoff* ” for the *t*-paired test or *p* binomial value (Carvalho et al., 2016). Finally, corrected *p*-value was calculated according to the Benjamin–Hochberg procedure (Carvalho et al., 2016). The final report of quantification, containing only proteins with an absolute fold change ≥ 1.5 and *p* ≤ 0.05, was exported to Microsoft Excel.

Ontological classification on cellular component, molecular function, and biological process was made through Gene Ontology database (http://geneontology.org/) using the Gene Ontology Explorer tool (GOEx) from the PatternLab (Carvalho et al., 2009). Interactome network analysis was mapped to KEGG pathways using the web-accessible program IIS- Integrated Interactome system (http://www.lge.ibi.unicamp.br/lnbio/IIS/) (Carazzolle et al., 2014). The enriched biological processes of the Gene Ontology database were calculated in each network using the hypergeometric distribution (Carazzolle et al., 2014). The interaction network was viewed using Cytoscape version 2.8.3 (http://www.cytoscape.org/).

### Signal Peptide, Alternative Secretion Pathway and Exosomal Origin Prediction

SignalP 4.1 server (http://www.cbs.dtu.dk/services/SignalP/) was used to predict the N-terminal signal peptide contained in classically secreted proteins (Petersen et al., 2011) and SecretomeP 2.0 server (http://www.cbs.dtu.dk/services/SecretomeP/) was used to predict non-classical *i.e*., not signal peptide triggered protein secretion (Bendtsen et al., 2004) from our identified proteins. The proteins positively predicted by one of the servers was considered as secreted. The database (Release date: 29 July 2015) from ExoCarta (http://www.exocarta.org/) was used to identify exosomal proteins previously reported for *M. musculus, Rattus novergicus*, or *Homo sapiens*.

### Measurement of Galectin-1 and Plasminogen by ELISA

Galectin-1 and plasminogen levels were measured in the thymic interstitial fluid by ELISA assays according to the manufacturer's procedures (Abcam Cambridge, UK, Cat. Ab119595 and ab197748). Levels of galectin-1 and plasminogen were expressed in pg/mL.

### Histological Analysis of the Thymus

Fragments of the thymus embedded in OCT compound medium (Sakura TissueTek) were cut into 5 mm thick sections and mounted on microscope slides. Sections of the thymus were stained with Mayer's hematoxylin and examined by light microscopy (Nikon Eclipse E400-Tokyo, Japan). Cortical and medullar areas of the thymus were evaluated under a light microscope and quantified in at least 5 fields at 100x magnification and another 5 fields at 400x using the ImageJ 1.48v software (NIH, USA). Cortex:medulla ratio was calculated by dividing the media of cortex area by the media of medulla region of each animal.

### Statistics

Statistical analysis was performed using two-way analysis of variance, ANOVA, with Bonferroni *post-hoc* test, or unpaired Student's *t*-test (GraphPad Prism version 6). Statistical significance was accepted at *p* < 0.05.

## Results

### Protein Malnutrition Alters the Thymic Microarchitecture in BALB/c Mice

**Figure 2:**
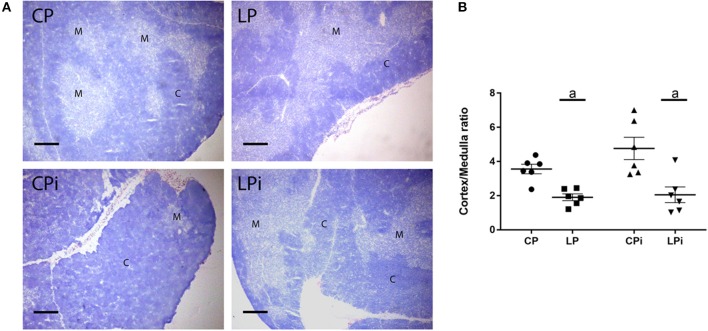
Thymic cortical and medullary regions of BALB/c mice submitted to protein restriction and infected with *L. infantum*. Thymic sections were stained by hematoxylin and cortical or medullary regions were quantified as described in Experimental section. **(A)** Representative images of thymic cortical and medullary regions in each experimental group; **(B)** Cortex:medulla ratio, bars represent mean ± SEM. Data are representative of two independent experiments with 6 animals per group. Magnification bar: 200 μm. C, cortex; M, medulla. CP: animals fed 14% protein diet, LP: animals fed 4% protein diet, CPi: animals fed 14% protein diet and infected, LPi: animals fed 4% protein diet and infected. Two-way ANOVA analysis with Bonferroni *post-hoc* test. Statistical differences due to diet: **a** (*p* < 0.0001).

Histopathological analysis of the thymus revealed changes in the cortical and medullar areas of the thymus of infected and/or malnourished animals (Figure 2A). Quantitation of cortical and medullar areas revealed that malnourished animals (LP and LPi) presented a significant reduction in the cortical area showing a cortical: medullar ratio of 1.9 and 2.0, respectively (Figure 2B). These values represent a reduction of 46 and 43% cortical area, respectively, when compared to the control group (CP - cortex:medulla ratio = 3.5). In contrast, the CPi animals presented an increase in the cortical area relative to medulla, and the cortical:medullar ratio is higher in this group when compared to the control group (CP). However, such difference was not statistically significant.

### Protein Malnutrition Alters Protein Abundance in the Thymic Interstitial Fluid in BALB/c Mice Infected With *L. infantum*

To gain insights into the effects of malnutrition in the thymic microenvironment during *L. infantum* infection, we used a quantitative proteomics approach for the identification of the global changes in the IF proteomes of malnourished BALB/c mice infected with *L. infantum*. We compared four groups of IF samples: animals fed 14% protein (iTRAQ labeled 114), animals fed 4% protein (iTRAQ labeled 115), animals fed 14% protein and infected with *L. infantum* (iTRAQ labeled 116), and animals fed 4% protein and infected with *L. infantum* (iTRAQ labeled 117).

In total, 3,098 peptides identified in the IF samples were assigned, with 1% FDR, to 280 proteins (Supplementary Table 1). Ninety percent (253/280) of the proteins were identified with at least two peptides and the remaining 10% (27/280) were accepted even with one peptide since they were unique or had more than one evidence of their presence in the sample (two or more spectra and/or two or more states of charge).

**Figure 3:**
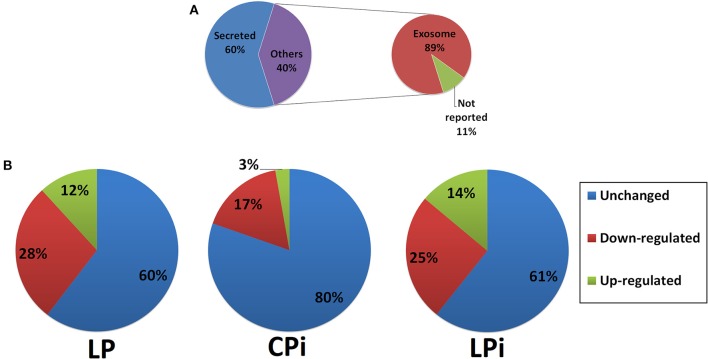
Proteomics analysis of thymic interstitial fluid. **(A)** Percentage of secreted or exosomal proteins identified in the thymic IF of BALB/c mice. **(B)** Percentages of differentially abundant proteins identified in the thymic IF of CPi, LP, or LPi animals relative to CP mice. LP: animals fed 4% protein diet, CPi: animals fed 14% protein diet and infected, LPi: animals fed 4% protein diet and infected.

Many secreted proteins identified in the IF of the thymus are secreted *bona fide* moieties, including albumin, coagulation factors, complement factors, carrier proteins and apolipoproteins (Supplementary Table 1). Using SignalP and Secretome P servers we identified which proteins are secreted following classical or alternative pathways (Supplementary Table 1). Only for 3% proteins (7/280) were predicted a signal peptide, i.e., they are secreted by the classical pathway exclusively; 43% proteins (121/280) were predicted to be secreted by an alternative pathway exclusively and 14% proteins (39/280) were predicted as presenting both classical and non-classically signals for secretion. In total, 60% (167/280) proteins were identified as secreted proteins. Interestingly, 127 out of these 167 proteins have also been reported as secreted by exosomes. The remaining 40% (113/280) proteins were not predicted as secreted by any of the servers used here; however, 89% (100/113) of them were previously reported as exosomal proteins and 11% (13/113) were neither predicted as exosomal nor secreted by classical or non-classical secretion pathways (Figure 3A and Supplementary Table 1). In total, 81% (227/280) proteins identified in the thymic IF are secreted by exosomes. Previously reported thymic exosomal proteins were identified in our samples, such as galectin-1, macrophage migration inhibitory factor and plasminogen (Simpson et al., 2009; Turiak et al., 2011; Skogberg et al., 2015). Eight proteins were annotated as exclusively intracellular and 10 proteins do not have subcellular location information yet.

Proteins from CP group were considered as the reference *status* and variations in protein abundance in the other conditions were evaluated against that group. In total, 112, 55, and 109 proteins showed significant differences in abundance at LP, CPi, and LPi animals, respectively, when compared to the CP group (Supplementary Figure 1).

Among the 112 proteins that significantly changed in LP mice, the relative abundance of 78 (28% of the total proteins identified, 78/280) was decreased whereas the relative abundance of 34 (12% of the total proteins identified, 34/280) was increased (Figure 3B and Supplementary Table 1). Thymic IF from CPi animals presented 55 proteins with altered abundance, 47 of which showed decreased relative abundance (17% of the total identified proteins, 47/280) and 8 increased (3% of the total identified proteins, 8/280) (Figure 3B and Supplementary Table 1). Differently, thymic IF from LPi mice showed significant changes in the abundance of 109 proteins. The relative abundance of 71 (25% of the total identified proteins, 71/280) was significantly decreased whereas the relative abundance of 38 (14% of the total identified proteins, 38/280) was increased (Figure 3B and Table 1).

**Table 1:** Differentially abundant proteins in the thymic interstitial fluid of malnourished BALB/c mice infected with *L. infantum* (LPi).

**ProtID**	**Daltons**	**Sequence count**	**Spectral count**	**Avg Log fold**	**Fold change**	**Stouffers *P* value**	**Description**	**Gene**	**Abundance relative to CP**
P56480	56247.46	1	2	1.581	4.9	0.011	ATP synthase subunit beta, mitochondrial	Atp5b	Down
P62830	14838.05	1	2	0.696	2.0	0.049	60S ribosomal protein L23	Rpl23	Down
Q9D0T1	14146.54	1	2	0.839	2.3	0.045	NHP2-like protein 1	Nhp2l1	Down
Q8K3C3	21505.1	1	2	0.719	2.1	0.035	Protein LZIC	Lzic	Down
Q91XV3	22055.58	1	2	0.945	2.6	0.023	Brain acid soluble protein 1	Basp1	Down
Q9CQQ8	11610.95	1	2	0.601	1.8	0.044	U6 snRNA-associated Sm-like protein LSm7	Lsm7	Down
P40142	67569.57	1	2	0.644	1.9	0.033	Transketolase	Tkt	Down
P60710	41691.72	1	2	0.988	2.7	0.029	Actin, cytoplasmic 1	Actb	Down
P68037	17832.23	1	2	0.71	2.0	0.010	Ubiquitin-conjugating enzyme E2 L3	Ube2l3	Down
Q8K1I7	50031.76	1	2	0.792	2.2	0.010	WAS/WASL-interacting protein family member 1	Wipf1	Down
P17751	32153.25	1	4	0.439	1.6	0.012	Triosephosphate isomerase	Tpi1	Down
Q9QXT0	20736.25	1	4	0.429	1.5	0.026	Protein canopy homolog 2	Cnpy2	Down
Q9DBP5	22133.28	1	4	0.504	1.7	0.010	UMP-CMP kinase	Cmpk1	Down
O08553	62220.58	1	4	0.443	1.6	0.026	Dihydropyrimidinase-related protein 2	Dpysl2	Down
P63158	24860.15	1	4	1.221	3.4	0.010	High mobility group protein B1	Hmgb1	Down
Q91WJ8	68479.01	1	4	1.2	3.3	0.010	Far upstream element-binding protein 1	Fubp1	Down
Q99KC8	87069.41	1	5	1.083	3.0	0.010	von Willebrand factor A domain-containing protein 5A	Vwa5a	Down
P30416	51521.93	1	5	1.07	2.9	0.010	Peptidyl-prolyl cis-trans isomerase FKBP4	Fkbp4	Down
Q9D8B3	24902.55	1	5	0.56	1.8	0.010	Charged multivesicular body protein 4b	Chmp4b	Down
Q9D1A2	52715.6	1	5	0.681	2.0	0.010	Cytosolic non-specific dipeptidase	Cndp2	Down
P16858	35769.2	1	5	0.705	2.0	0.010	Glyceraldehyde-3-phosphate dehydrogenase	Gapdh	Down
Q6NZB0	29776.36	1	5	0.915	2.5	0.010	DnaJ homolog subfamily C member 8	Dnajc8	Down
P32067	47708.95	1	5	1.558	4.7	0.010	Lupus La protein homolog	Ssb	Down
P50543	11057.48	1	6	0.92	2.5	0.010	Protein S100-A11	S100a11	Down
P63038	60899.38	1	6	1.054	2.9	0.035	60 kDa heat shock protein, mitochondrial	Hspd1	Down
P18760	18529.67	1	6	0.949	2.6	0.010	Cofilin-1	Cfl1	Down
P62774	12834.61	1	7	0.607	1.8	0.010	Myotrophin	Mtpn	Down
P99026	29079.32	1	7	0.653	1.9	0.010	Proteasome subunit beta type-4	Psmb4	Down
Q9JL35	45298.7	1	7	0.746	2.1	0.010	High mobility group nucleosome-binding domain-containing protein 5	Hmgn5	Down
P60335	37455.93	1	7	1.203	3.3	0.010	Poly(rC)-binding protein 1	Pcbp1	Down
P11031	14400.41	1	8	0.781	2.2	0.010	Activated RNA polymerase II transcriptional coactivator p15	Sub1	Down
Q99PT1	23374.8	1	8	0.605	1.8	0.010	Rho GDP-dissociation inhibitor 1	Arhgdia	Down
Q9CQR2	9117.54	1	8	0.801	2.2	0.010	40S ribosomal protein S21	Rps21	Down
O35685	38316.28	2	6	1.113	3.0	0.008	Nuclear migration protein nudC	Nudc	Down
Q05144	21409.07	2	7	1.799	6.0	0.006	Ras-related C3 botulinum toxin substrate 2	Rac2	Down
P28667	20135.44	2	7	1.17	3.2	0.008	MARCKS-related protein	Marcksl1	Down
Q62418	48651.6	2	8	0.644	1.9	0.005	Drebrin-like protein	Dbnl	Down
P19157	23576.1	2	8	0.451	1.6	0.006	Glutathione S-transferase P 1	Gstp1	Down
P63028	19431.54	2	8	2.293	9.9	0.007	Translationally-controlled tumor protein	Tpt1	Down
Q6IRU2	28432.42	2	9	1.843	6.3	0.008	Tropomyosin alpha-4 chain	Tpm4	Down
P09405	76658.76	2	9	1.444	4.2	0.007	Nucleolin	Ncl	Down
P00920	28996.49	2	10	0.662	1.9	0.008	Carbonic anhydrase 2	Ca2	Down
Q9JMG1	16340.9	2	11	1.952	7.0	0.005	Endothelial differentiation-related factor 1	Edf1	Down
P62869	13143.64	2	12	0.444	1.6	0.006	Transcription elongation factor B polypeptide 2	Tceb2	Down
P09411	44503.98	2	13	1.268	3.6	0.013	Phosphoglycerate kinase 1	Pgk1	Down
O08997	7315.65	2	13	0.499	1.6	0.005	Copper transport protein ATOX1	Atox1	Down
Q9CQM5	13987.73	2	14	0.738	2.1	0.009	Thioredoxin domain-containing protein 17	Txndc17	Down
P99024	49620.96	2	15	1.344	3.8	0.005	Tubulin beta-5 chain	Tubb5	Down
P27773	56624.67	3	15	0.521	1.7	0.003	Protein disulfide-isomerase A3	Pdia3	Down
P05784	47491.21	3	16	1.583	4.9	0.004	Keratin, type I cytoskeletal 18	Krt18	Down
Q91VW3	10452.26	3	20	0.744	2.1	0.002	SH3 domain-binding glutamic acid-rich-like protein 3	Sh3bgrl3	Down
O35381	28502.23	3	20	0.863	2.4	0.002	Acidic leucine-rich nuclear phosphoprotein 32 family member A	Anp32a	Down
O88569	37361.71	3	23	0.568	1.8	0.002	Heterogeneous nuclear ribonucleoproteins A2/B1	Hnrnpa2b1	Down
O70251	24660.23	3	24	0.689	2.0	0.002	Elongation factor 1-beta	Eef1b	Down
Q3U0V1	76709.7	4	18	0.985	2.7	0.001	Far upstream element-binding protein 2	Khsrp	Down
P28665	165174.53	4	18	1.176	3.2	0.001	Murinoglobulin-1	Mug1	Down
P34022	23563.7	4	21	0.832	2.3	0.001	Ran-specific GTPase-activating protein	Ranbp1	Down
Q9WVA4	22363.15	4	23	1.251	3.5	0.001	Transgelin-2	Tagln2	Down
Q6ZWZ6	14487.47	4	26	1.08	2.9	0.002	40S ribosomal protein S12	Rps12	Down
P99027	11625.82	4	26	1.736	5.7	0.001	60S acidic ribosomal protein P2	Rplp2	Down
P11679	54513.45	4	27	1.036	2.8	0.010	Keratin, type II cytoskeletal 8	Krt8	Down
P16045	14838.19	4	32	0.729	2.1	0.001	Galectin-1	Lgals1	Down
P63101	27735.73	5	23	1.264	3.5	0.001	14-3-3 protein zeta/delta	Ywhaz	Down
P10639	11649.63	5	37	1.136	3.1	0.000	Thioredoxin	Txn	Down
P16015	29329.63	6	39	0.826	2.3	0.000	Carbonic anhydrase 3	Ca3	Down
E9PZF0	30162.67	6	41	1.074	2.9	0.000	Nucleoside diphosphate kinase	Gm20390	Down
Q61599	22818.48	7	41	0.859	2.4	0.000	Rho GDP-dissociation inhibitor 2	Arhgdib	Down
P48036	35712.2	7	51	0.585	1.8	0.000	Annexin A5	Anxa5	Down
P26645	29625.81	7	52	1.267	3.6	0.000	Myristoylated alanine-rich C-kinase substrate	Marcks	Down
P07759	46831.98	10	61	1.296	3.7	0.000	Serine protease inhibitor A3K	Serpina3k	Down
Q01853	89247.71	12	52	1.026	2.8	0.000	Transitional endoplasmic reticulum ATPase	Vcp	Down
Q63918	46717.37	1	2	−0.903	2.5	0.036	Serum deprivation-response protein	Sdpr	Up
P10493	136432.48	1	2	−1.186	3.3	0.041	Nidogen-1	Nid1	Up
Q6P8J7	47425.31	1	2	−0.925	2.5	0.047	Creatine kinase S-type, mitochondrial	Ckmt2	Up
Q8BMF4	67880.67	1	2	−0.97	2.6	0.020	Pyruvate dehydrogenase complex component E2	Dlat	Up
Q8BKZ9	53947.22	1	2	−0.429	1.5	0.022	Pyruvate dehydrogenase protein X component, mitochondrial	Pdhx	Up
P37804	22543.37	1	2	−0.666	1.9	0.016	Transgelin	Tagln	Up
O55042	14458.17	1	2	−0.602	1.8	0.047	Alpha-synuclein	Snca	Up
P20918	90730.9	1	2	−0.983	2.7	0.036	Plasminogen	Plg	Up
Q61425	34423.87	1	3	−1.349	3.9	0.010	Hydroxyacyl-coenzyme A dehydrogenase, mitochondrial	Hadh	Up
P06151	36457.21	1	4	−0.616	1.9	0.010	L-lactate dehydrogenase A chain	Ldha	Up
P31786	9976.13	1	4	−0.415	1.5	0.014	Acyl-CoA-binding protein	Dbi	Up
P35505	46128.03	1	5	−0.607	1.8	0.036	Fumarylacetoacetase	Fah	Up
P56375	11852.05	1	6	−0.644	1.9	0.010	Acylphosphatase-2	Acyp2	Up
P09528	21035.25	1	6	−0.548	1.7	0.018	Ferritin heavy chain	Fth1	Up
Q9CR68	29331.19	1	7	−0.557	1.7	0.010	Cytochrome b-c1 complex subunit Rieske, mitochondrial	Uqcrfs1	Up
Q91XL1	37389.49	1	7	−0.458	1.6	0.024	Leucine-rich HEV glycoprotein	Lrg1	Up
Q91V76	34955.33	1	8	−0.641	1.9	0.010	Ester hydrolase C11orf54 homolog	C11orf54	Up
P99028	10409.97	1	8	−0.836	2.3	0.010	Cytochrome b-c1 complex subunit 6, mitochondrial	Uqcrh	Up
Q9WTP6	26433.69	1	8	−0.491	1.6	0.010	Adenylate kinase 2, mitochondrial	Ak2	Up
P08228	15914.79	1	16	−0.721	2.1	0.010	Superoxide dismutase [Cu-Zn]	Sod1	Up
Q99JY0	51335.4	2	5	−1.348	3.8	0.005	Trifunctional enzyme subunit beta, mitochondrial	Hadhb	Up
P52503	12993.66	2	5	−0.574	1.8	0.008	NADH dehydrogenase [ubiquinone] iron-sulfur protein 6, mitochondrial	Ndufs6	Up
Q9D2G2	48945.47	2	12	−0.773	2.2	0.006	2-oxoglutarate dehydrogenase complex component E2	Dlst	Up
Q6LD55	11293.81	2	14	−0.727	2.1	0.005	APOAII	Apoa2	Up
Q61171	21747.05	2	14	−0.495	1.6	0.006	Peroxiredoxin-2	Prdx2	Up
Q3THE6	20664.39	2	15	−0.757	2.1	0.005	Ferritin	Fth	Up
Q8BWT1	41785.43	2	15	−0.812	2.3	0.005	3-ketoacyl-CoA thiolase, mitochondrial	Acaa2	Up
P21614	53546.98	2	17	−0.499	1.6	0.009	Vitamin D-binding protein	Gc	Up
P97807	54304.06	3	10	−0.678	2.0	0.003	Fumarate hydratase, mitochondrial	Fh	Up
P14152	36470.07	3	13	−0.814	2.3	0.003	Malate dehydrogenase, cytoplasmic	Mdh1	Up
Q99LC5	34969.49	3	16	−0.768	2.2	0.003	Electron transfer flavoprotein subunit alpha, mitochondrial	Etfa	Up
P56391	10046.87	3	16	−0.779	2.2	0.003	Cytochrome c oxidase subunit 6B1	Cox6b1	Up
Q8BH95	31436.2	4	21	−1.269	3.6	0.001	Enoyl-CoA hydratase, mitochondrial	Echs1	Up
P08249	35570.75	4	22	−0.697	2.0	0.001	Malate dehydrogenase, mitochondrial	Mdh2	Up
Q99KI0	85392.01	7	33	−0.785	2.2	0.000	Aconitate hydratase, mitochondrial	Aco2	Up
Q91X72	51267.17	8	50	−0.418	1.5	0.000	Hemopexin	Hpx	Up
Q9DCW4	27587.97	8	51	−1.378	4.0	0.000	Electron transfer flavoprotein subunit beta	Etfb	Up
Q921I1	76655.71	12	87	−0.66	1.9	0.000	Serotransferrin	Tf	Up

*Difference in protein abundance is relative to control mice (CP group)*.

**Figure 4:**
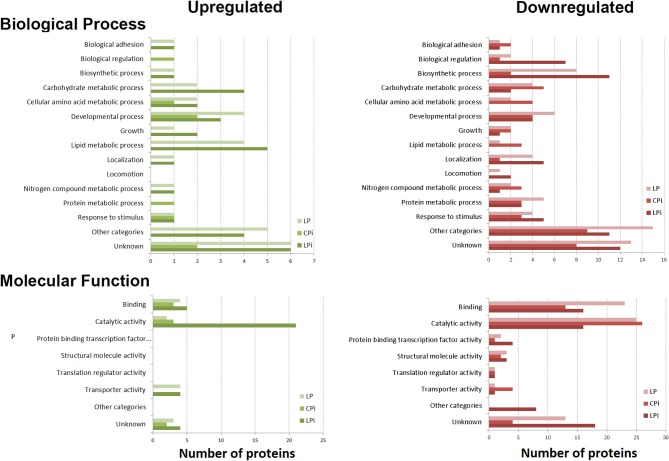
Biological and molecular processes associated with altered protein abundance in the thymic IF of BALB/c mice. Functional annotation of biological process and molecular function assigned to the differentially abundant proteins as annotated in the Gene Ontology database. Differential abundance in LP, CPi, or LPi mice, relative to CP animals. LP: animals fed 4% protein diet, CPi: animals fed 14% protein diet and infected, LPi: animals fed 4% protein diet and infected.

Differentially abundant proteins were classified according to the functional groups belonging to two main categories of gene ontology: *biological process* and *molecular function*. The highest number of positively regulated proteins in malnourished and infected animal samples was associated with *lipid and amino acid metabolism* and *developmental processes*, whereas negatively regulated proteins were mainly involved in *biosynthetic processes, biological regulation, and locomotion*. Many of the positively or negatively regulated proteins were associated with *catalytic and binding activities*. Some of the positively regulated proteins were classified into the group *transporter activity* whereas some negatively regulated proteins were associated with *binding protein activity to transcription factors*. These results are summarized in Figure 4.

### Galectin-1 Is Significantly Decreased Whereas Plasminogen Is Increased in Malnourished-Infected Mice

**Figure 5:**
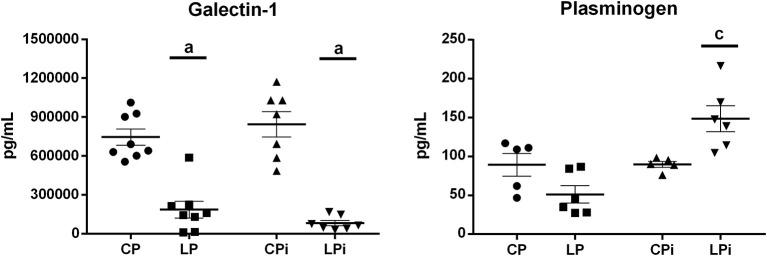
ELISA quantification of proteins secreted into the thymic IF. Galectin-1 and plasminogen were quantified by a commercial ELISA kit and their values are expressed in pg/mL (*n* = 5–8 animal per group). Statistical differences due to diet: **a** (*p* < 0.0001), and interaction between diet and infection: **c** (*p* < 0.05) were determined using two-way ANOVA with Bonferroni *post-hoc* test. CP: animals fed 14% protein diet, LP: animals fed 4% protein diet, CPi: animals fed 14% protein diet and infected, LPi: animals fed 4% protein diet and infected.

In order to further verify, by an alternative method, the differences in protein abundance observed in the thymic IF by iTRAQ, galectin-1 (downregulated in malnourished mice) and plasminogen (upregulated in malnourished mice) were selected to be quantified in the thymic IF using commercial ELISA kits. Galectin-1 is a β-galactoside binding protein secreted by TECs that modulates crucial biological processes in thymus, such as cell adhesion, cell-cell interaction, migration, proliferation, and apoptosis of thymocytes (Baum et al., 1995; Perillo et al., 1998; Camby et al., 2006; Stillman et al., 2006). Plasminogen is the zymogen form of plasmin, a serine peptidase involved in fibrinolytic activities, which substrates include fibronectin, laminin, and von Willebrand factor, among others (Liotta et al., 1981). In the thymus, plasminogen may have an important role in tissue remodeling. In agreement with our proteomics data, analyses by ELISA revealed that the levels of galectin-1 were significantly reduced by malnutrition in the LP and LPi animals, relative to the CP group, whereas plasminogen levels were significantly increased in the thymic IF of malnourished and infected animals (LPi), in relation to the CP group (Figure 5).

### Proteins Associated With Beta Oxidation of Fatty Acids or the Tricarboxylic Acid Cycle Are Significantly Increased in Malnourished Mice Infected With *L. infantum*

To explore which functions of IF proteins were altered by protein malnutrition and *L. infantum* infection we performed a functional pathway analysis using the IIS- interactome system web-accessible software. Interaction networks among differentially abundant proteins in each group were built to analyze the enriched biological processes shared by quantified proteins.

Proteins with altered abundance in the thymic IF of CPi animals were mostly less abundant when compared to the CP group (Supplementary Figure 2A). Groups of negatively regulated proteins that were better defined included: *tricarboxylic acid cycle* (FH, DLST, MDH1, MDH2) and *negative regulation of the apoptotic process* (PRDX3, NPM1, HSPD1, GSTP1). In addition, the proteins YWHAZ, NPM1, HSPD1, and VCP were also found with decreased relative abundance in the CPi group.

The LP group displayed two well-defined clusters of upregulated proteins: proteins associated with *fatty acid beta-oxidation* (HADHB, ACAA2, ECHS1); and proteins associated with the *tricarboxylic acid cycle* (DLST, DLAT, ACO2, PDHB). On the other hand, we observed several clusters of proteins with reduced abundance in LP animals: *mRNA processing* (PCPBP1, NHP2L1, KHSRP, LSM7, HNRNPA2B1); *cellular redox homeostasis* (TXN, PDIA3, SH3BGRT3); *oxidative stress response* (PRDX2, PEBP1, CA3, ATOX1); *drug response* (YWHAZ, DPYSL2, PEBP1); *transcription* (DPY30, ANP32A, CBX3); *metabolic process glutamate* (GOT1, GLUT); among others (Supplementary Figure 2B).

**Figure 6:**
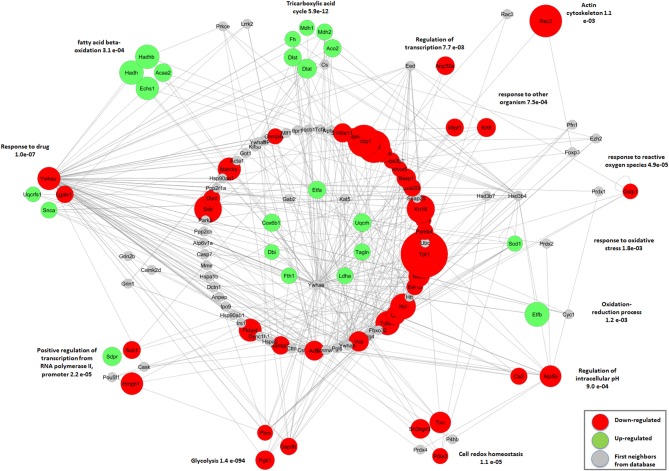
Functional interaction network among differentially abundant proteins identified in the thymic IF of LPi mice grouped by biological process. The proteins with differential abundance in the LPi group relative to the CP group were grouped according to the biological process enriched with *p* < 0.05. The proteins represented in the central circles did not present clusters with significant enrichment in the biological process. Network was built using the IIS (Integrated interactome system) platform and viewed in Cytoscape software version 2.8.3.

Similar to LP animals, the thymic IF of LPi mice exhibited two clusters of positively regulated proteins: (i) proteins associated with *beta oxidation of fatty acids* (HADH, HADHB, ACAA2, ECHS1) and (ii) proteins associated with the *tricarboxylic acid cycle* (DLST, DLAT, ACO2, FH, MDH1, MDH2) (Figure 6). A positive regulation was also observed in the group of *iron cellular homeostasis* (FH, FTH1). Several clusters of proteins whose abundance was reduced were evident in the network: *mRNA processing* (PCBP1, NHP2L1, LSM7, KHSRP, HNRNPA2B1); *protein ubiquitination* (VCP, UBE2I3, TCEB2); *cellular redox homeostasis* (TXN, PDIA3, SH3BGRT3); *glycolysis* (TPI1, PGK1); and *negative regulation of apoptotic processes* (KRT18, GSTP1). In addition, the biological process group termed *drug response* clustered both increased (UQCRFS1, SNCA, SOD1) and decreased proteins (YWHAZ, LGALS1, DPYSL2). Similar to the LP and CPi group, YWHAZ and VCP proteins were found with diminished abundance.

Although, LP and LPi show similar behaviors regarding the alteration of abundance of various groups of proteins, the LPi animals exhibit additional changes that were not observed in the LP group (Supplementary Table 2). For example, LPi animals exhibited decrease in the abundance of proteins involved in *negative regulation of apoptotic processes* that were not significantly altered in the LP group. Interestingly, this cluster was also significantly decreased in CPi group suggesting that infection *per se* is also modulating the abundance of proteins in LPi mice. In addition, LPi animals exhibited, for example, significant increase of *creatine kinase S-type* protein whereas LP animals showed decreased abundance of this protein (Supplementary Table 2).

Comparison between proteomics data from CPi and LPi animals also revealed that the eight proteins upregulated in CPi mice were not upregulated in LPi animals and three of them were even downregulated. For example, proteins that increased in response to infection in CPi animals, such as *microtubule-associated protein 4* and *thioredoxin domain-containing protein 17* (Supplementary Table 2) were unchanged or even decreased, respectively, in the LPi animals. In addition, infection in CPi induced downregulation of 47 proteins but 19 of them were upregulated by malnutrition in LPi. For instance, CPi animals showed a 6.7-fold reduction of *vitamin D-binding protein* whereas in LPi mice this protein was significantly upregulated (1.6-fold change). Those data emphasize that changes in protein abundance due to a precedent malnutrition could affect the immune response to infection.

### Protein Malnutrition Decreases the Proliferation of Single Positive and Double Positive T Cells From the Thymus of *L. infantum*-Infected Mice

**Figure 7:**
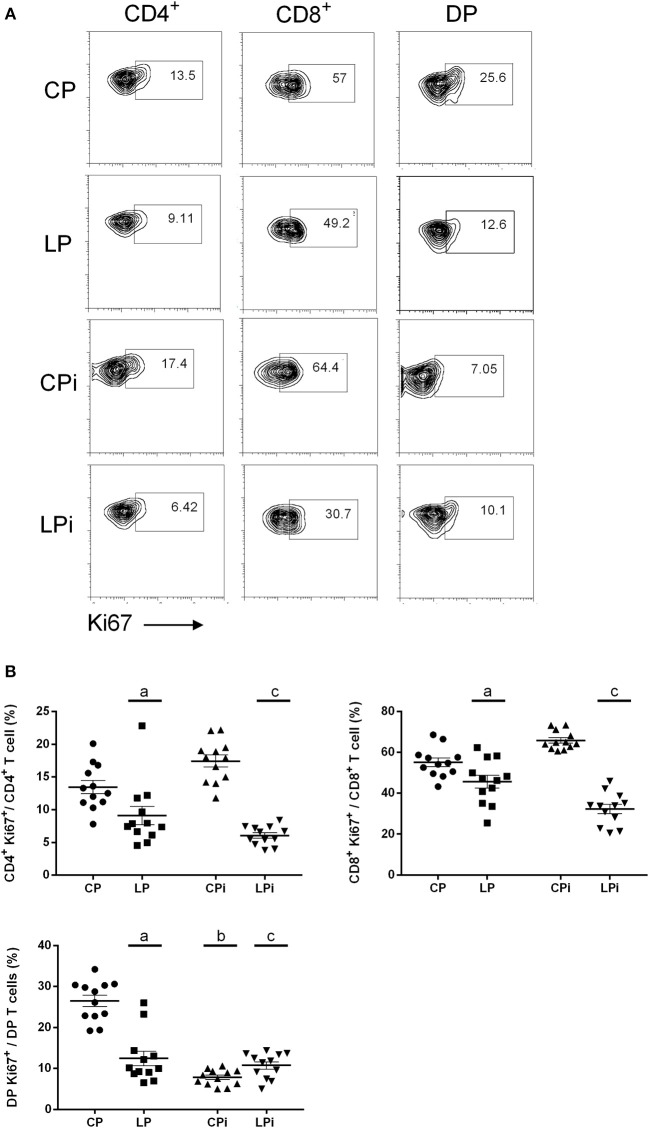
Flow cytometry analysis of T cell subpopulations proliferating in the thymus of malnourished animals infected with *L. infantum*. Lymphocyte subpopulations from the thymus of the experimental groups were measured using FACS analysis as described in the Materials and Methods. **(A)** Representative plots of thymocyte subpopulations stained with Ki67. All images shown were selected from one single experiment and are representative of the phenotype obtained at each group; numbers indicate the percentages of the corresponding gate shown. Displacement of some subpopulations of thymocytes was observed during the analyzes and an adjust of the Ki67+ gate was necessary to distinguish proliferative and non-proliferative cells. The gates were defined using the same criteria described in Supplementary Figure 4. **(B)** Percentage of thymocytes subsets expressing Ki67 (*n* = 12 animals per group from two independent experiments with 6 animals each). CP: animals fed 14% protein diet; LP: animals fed 4% protein diet, CPi: animals fed 14% protein diet and infected; LPi: animals fed 4% protein diet and infected. Two-way ANOVA analysis with Bonferroni *post-hoc* test. Statistical differences due to diet: **a** (*p* < 0.001), infection: **b** (*p* < 0.001) and interaction between diet and infection: **c** (*p* < 0.001).

As proteomics data were suggestive of a non-proliferative thymic microenvironment, we evaluated the expression of Ki67 on T cell subsets from the thymus of malnourished animals infected with *L. infantum*. Flow cytometry analysis revealed that malnutrition impaired the proliferative capabilities of thymic lymphocyte subpopulations, altering the percentage of proliferative thymocytes (Figure 7A and Supplementary Figure 4). The percentage of proliferative (Ki67^+^) single positive T cells (CD4^+^ or CD8^+^ T cells) was significantly decreased in the thymus of malnourished animals (*p* < 0.001). Infection with *L. infantum* in malnourished animals induced a still more accented decrease in the percentage of CD4^+^Ki67^+^ and CD8^+^Ki67^+^ T cells (*p* < 0.001). By contrast, CPi mice displayed a trend to increase the percentage of CD4^+^ and CD8^+^ T cells undergoing proliferation. Both independently and by interaction, protein malnutrition and infection induced a significant decrease in the percentage of double positive T cells (CD4^+^CD8^+^ T cells, DP) proliferating and that had recently proliferated, based on Ki67 expression, in the thymus of BALB/c mice (*p* < 0.001) (Figure 7B).

## Discussion

We previously demonstrated that interaction between protein malnutrition and *L. infantum* infection in BALB/c mice resulted in significant thymic atrophy, hypocellularity, changes in T cell subsets and thymic infection (Cuervo-Escobar et al., 2014; Losada-Barragan et al., 2017). These events were accompanied by significant alterations in the gene expression, at mRNA and protein levels, of chemokines, and adhesion molecules involved in T cell migration whereas mRNA and protein levels of proapoptotic molecules were not affected (Cuervo-Escobar et al., 2014; Losada-Barragan et al., 2017). All those alterations accelerated the pathological events observed during the course of infection with *L. infantum* resulting in an increased parasite load in spleen, without infection resolution in the liver, and an earlier drastic damage of splenic architecture (Cuervo-Escobar et al., 2014; Losada-Barragan et al., 2017). Such observations led us to suggest that instead of increasing T cell apoptosis, malnutrition seriously affected the migration of these cells (both central and peripheral migration), leading to defects in T cell-mediated immune responses to *L. infantum*. Remarkably, *ex vivo* assays revealed that thymocytes from LPi group stimulated with chemotactic stimuli did preserve their migratory capabilities, indicating that in malnourished mice the thymic microenvironment that mediates cell mobilization, including extracellular matrix molecules and protein milieu, are altered rather than the migratory capabilities of T cells *per se* (Cuervo-Escobar et al., 2014; Losada-Barragan et al., 2017). In the current study, we analyzed the thymus microarchitecture, the proteins circulating in the thymic microenvironment and the proliferation of thymocytes from malnourished BALB/c mice infected with *L. infantum* with the aim to identify the factors that could be influencing the pathophysiology of the organ in these conditions.

The thymic physiology is highly dependent of an ordered process for T-cell development, involving sequential migration of immature thymocytes through differentiated regions of the thymus that contribute for each cell to receive the proper signals and favor cell–cell interactions in a precise order and in a suitable microenvironment (Petrie and Zuniga-Pflucker, 2007). The thymic cortex is the location where β-selection and positive and negative selection occur and is mainly constituted by double negative (DN) and double positive (DP) T cell populations, whereas the thymic medulla is enriched of single positive (SP) CD4^+^ and CD8^+^ T cells expressing high levels of the αβTCR (Anderson et al., 2014). In this study, histopathological analysis of thymic microarchitecture of malnourished BALB/c mice infected or not with *L. infantum* revealed a significant ~2-fold reduction of the cortex:medulla ratio. Remarkably, this reduction was accompanied by a significant ~5-fold decrease in the abundance of cytokeratins K8 and K18, which are produced by TECs. These results could suggest that in malnourished mice (i) immune responses are not properly recruited to control thymic infection (Cuervo-Escobar et al., 2014; Losada-Barragan et al., 2017 and/or (ii) maturation and migration of T cells are not properly occurring in these animals in response to local and systemic infection. In fact, we observed that malnutrition drastically alters cellularity in infected animals, and total numbers of DP and SP T cells are significantly diminished in malnourished mice [(Cuervo-Escobar et al., 2014) and this study, Supplementary Figure 3]. During infection with other pathogens, it has been observed that recruiting of peripheral T cells back to the thymus has an important role in preventing the emergence and export of microorganism-tolerant T cells (Nobrega et al., 2013; Nunes-Alves et al., 2013). As we detected parasites in thymus of infected mice, it is possible to suggest that peripheral T cells reentry to the organ to control local infection; however, in malnourished animals, such responses could be altered. In addition, we previously reported a significant reduction in total DP (Cuervo-Escobar et al., 2014) and a significant increase of CCR7 expression in the thymus of LPi animals (Losada-Barragan et al., 2017). Thus, our results suggest that impairment of cortical area expansion due to malnutrition during *L. infantum* infection could alter key thymic processes such as (i) β-selection, (ii) proliferation, (iii) positive selection, and/or (iv) proper migration of DN and DP T cell populations, all of which occur in the cortex. In addition, our results also suggest that such alterations should be accompanied by dysregulations in distribution and location of ECM components that are essential to guide thymocyte differentiation and to drive the migration of thymocytes in the complexes with epithelial cells (Savino et al., 2000, 2004). However, such hypotheses remain to be further explored.

To analyze if the changes in the cortex:medulla ratio are accompanied by alterations in the levels of proteins secreted into the ECM milieu, composing a scenario where both structural and soluble factors would be affected by malnutrition, we performed a quantitative proteomics profiling of the thymic microenvironment for identification and quantification of proteins in IF samples. To our knowledge, this is the first quantitative proteomics study of the thymic IF and the first work showing the proteome changes occurring in the interstitial microenvironment of the thymus during interaction of two pathological conditions: protein malnutrition and *L. infantum* infection.

In total, we identified 280 proteins in thymic IF, and most of them (97%) are secreted by some predicted mechanism either exosomal, classical or alternative. Remarkably, we identified 81% proteins previously reported as exosomal. In general, exosomes and other secreted microvesicles typically carry ubiquitously expressed molecules, such as intracellular metabolic enzymes, cytoskeletal proteins, chaperones, and ribosomal proteins (Shin et al., 2009; Zhu et al., 2015). Exosomes are specifically enriched in molecules associated with their biogenesis or in molecules selectively packaged within them (Coakley et al., 2015). These proteins may constitute more than half of the secreted proteins as it has been observed in cancer cells (Wu et al., 2008). Despite our aim was not to characterize exosomes or extracellular vesicles, by comparison with previous studies reported elsewhere (Simpson et al., 2008; Skogberg et al., 2013, 2015; Lundberg et al., 2016) allow us to state that our IF samples contained proteins from exosomal origin. In fact, our proteomics data include typical exosomal markers and proteins often identified in exosomes such as: actin cytoplasmic 1 (ACTB), annexin A5 and 6 (ANXA5, ANXA6), cofilin-1 (CFL1), alpha-enolase (ENO1), phosphoglycerate kinase 1 (PGK1), pyruvate kinase PKM (PKM2), 14-3-3 protein zeta/delta (YWHAZ), serum albumin (ALB), L-lactate dehydrogenase A chain (LDHA), elongation factor 1-alpha 1 (EEF1A1), glyceraldehyde-3-phosphate dehydrogenase (GAPDH), and fructose-bisphosphate aldolase A (ALDOA) (http://www.exocarta.org/exosome_markers) (Simpson et al., 2009). Vesicles with hallmarks of exosomes have been described and characterized from primary cultures of thymic epithelial cells (TECs) (Simpson et al., 2009; Skogberg et al., 2013, 2015). Forty two percent of the proteins identified here were previously described as secreted by exosomes from TECs and/or in thymus of C57BL/6 mice (Skogberg et al., 2013, 2015; Lundberg et al., 2016). We also identified typical proteins with a known expression in TECs such as cytokeratins K8 and K18 (Lundberg et al., 2016). These data indicate that proteins identified in the thymic IF have a mixed origin, with a significant proportion being secreted by TECs.

Several studies have shown that exosomes are capable of presenting antigens for T cells by themselves or indirectly via uptake by dendritic cells (DCs) (Raposo et al., 1996). Exosomes have several functions in the activation and suppression of immune cells (Montecalvo et al., 2012; Deng et al., 2013) and are also proposed to play a role in the development of diseases and tissue homeostasis (Valadi et al., 2007; Pegtel et al., 2010; Aswad et al., 2014; Ridder et al., 2014; Banfai et al., 2019). In the thymus, secreted exosomes from stromal medullary epithelial cells (mTECs) can contribute both to intercellular antigenic transfer by spreading antigens within the thymus and to the thymic physiology maintenance (Skogberg et al., 2015; Lundberg et al., 2016). Our proteomics dataset reveals exosomal secretion of different types of proteins mainly related with protein expression regulation and metabolism, suggesting the presence of intercellular transfer mechanisms that go beyond antigen presentation and could include further thymic cellular functions. Thus, our data provide molecular bases to hypothesize that secretion of proteins via exosomes is an important mechanism of cell communication in the thymic microenvironment. However, such hypothesis remains to be further explored.

Considering that its content is specific, it is possible to suggest that changes in the abundance of different types of exosomal proteins could reflect the communication that is happening among the thymus cells under pathological conditions. We found that the thymic IF of low protein diet groups (LP and LPi) particularly displayed down-regulation of proteins associated with transcription, translation, mRNA processing, and proteolysis, while the CPi group presented few differential changes of these kind of proteins. These findings highlight a dysregulation of the protein expression in thymic cells and consequently in the thymic microenvironment physiology. Thus, the reduced abundance of those type of proteins due to low protein diet may alter the regulation of the proteins that are going to be transcribed, translated and degraded during infection condition, reducing the local and systemic response to *L. infantum* infection.

Galectin-1 (LGALS1) is an exosomal protein with crucial role in thymus functionality (Perone et al., 2006); it is produced by TECs and binds to thymocytes modulating the interaction strength to the TCR, and therefore influencing its selection (Baum et al., 1995). Galectin-1 plays a critical role in apoptosis of negatively selected thymocytes (Perillo et al., 1998). Furthermore, LGALS1 mediates leukemic cell differentiation (Zhao et al., 2011) and has an important role in the adhesion of different cell types to the ECM via the cross-linking of glycoproteins with ECM components such as laminin and fibronectin (Gameiro et al., 2010). Alterations in LGALS1 production lead to aberrant thymic selection and altered T cell populations (Liu et al., 2008). Remarkably, we detected diminished levels of LGAS1 in the IF of the thymus of LPi mice. Since Gal-1 promotes the apoptosis of immature cortical thymocytes *in vitro* (Perillo et al., 1998), it is possible to suggest that diminished levels of this protein within the thymic microenvironment of malnourished mice could have a deleterious impact in the processes of positive and/or negative selection. Interestingly, LPi mice also exhibited increased levels of antiapoptotic markers (Losada-Barragan et al., 2017). These results, together with the observation that protein malnutrition modified the cortex:medulla ratio in the thymus of *L. infantum* infected mice, reinforces the hypothesis that an abnormal interaction of thymocytes with ECM components occurs in malnourished mice. Such defect would imply altered signalization during T cell differentiation and defective thymocyte selection in these animals. On the other hand, we observed a significant increase of plasminogen in malnourished mice. As this protein is involved in tissue remodeling, its increased abundance suggests a potential role in the alteration of the thymic microarchitecture in those animals. Interestingly, increase of plasminogen was accompanied by a decrease in von Willebrand factor, a known substrate for plasmin, reinforcing the hypothesis that plasminogen is actively participating in remodeling the thymic microenvironment of malnourished mice.

Thymic secreted proteins such as Rho GDP-dissociation inhibitor 1 and 2 (Rho GDIa and GDIb, respectively) have an important role in T cell development and egress (Ishizaki et al., 2006). Deficiency in Rho GDIa and GDIb is involved in defective intrathymic differentiation and T cell migration, particularly exported from the thymus (Ishizaki et al., 2006). In agreement with previous reports (Lundberg et al., 2016), we observed that these proteins are secreted in the thymus by exosomes. Moreover, these proteins were downregulated in malnourished mice, suggesting that thymocyte differentiation and T cell egress might be compromised in those animals. As a consequence, a reduction in peripheral T cell population, as observed previously (Cuervo-Escobar et al., 2014), might have a deleterious impact on parasite control. Together, our results show that malnutrition alters abundance of proteins involved in thymic remodeling, thymocyte adhesion and differentiation, and T cell migration, all of which contribute to thymic atrophy and defective peripheral T cell colonization.

Although available molecular data of thymic metabolism are relatively scarce, studies on cancer cell metabolism have helped to understand the metabolic requirements of proliferating cells, and have facilitated their study on T cells. In particular, studies on the bioenergetic profile of T cells have revealed that their metabolism change dynamically with the state of activation and differentiation (Wang and Green, 2012; Pearce et al., 2013; Lochner et al., 2015; Buck et al., 2016; Rambold and Pearce, 2018). Consistent with the metabolism of other non-proliferative cells, naive T cells and memory T cells maintain low glycolysis rates and predominantly oxidize pyruvate derived from glucose through oxidative phosphorylation (OxPhos) or involving fatty acids oxidation (FAO) to generate ATP (Pearce et al., 2013). Following activation, T cells switch to an anabolic growth program and accumulation of biomass to generate daughter cells; a process that requires a greater demand for ATP and metabolic resources. In addition, catabolic ATP generation pathways such as β-oxidation of fatty acids are actively suppressed (Wang and Green, 2012). However, it is unknown which metabolic pathways are involved in the homeostasis of T cell proliferation and differentiation in the thymus. In this work, we found increased abundance of enzymes that participate in the catabolism of fatty acids via β-oxidation in malnourished animals. Enzymes such as enoyl-CoA hydratase (ECHS1), hydroxyl-coenzyme A dehydrogenase (HADH), 3-ketoacyl-CoA thiolase (ACAA2), and trifunctional beta subunit enzyme (HADHB) were significantly increased in that group. In addition, a high proportion of mitochondrial proteins participating in OxPhos and TCA were identified with greater abundance in malnourished (LP and LPi) mice in relation to CP animals. Such metabolic pattern seems to resemble a naïve or memory T cell metabolic profile and fits with a non-proliferative cell profile under malnutrition conditions in the thymus. Remarkably, such profiling is supported by the observation of a significant decrease in the percentage of proliferative SP and DP T cells in malnourished animals. Such results would also be in agreement with the microarchitecture alterations in the thymus of malnourished animals.

These findings could also be related with a diminished recirculation of peripheral T cells into the thymus and/or a defective proliferation and differentiation of thymocytes during protein malnutrition and *L. infantum* infection. In agreement, our results from flow cytometry analysis corroborated defective proliferation of SP and DP T cells in malnourished animals. Moreover, it is well-documented that during thymus infection by *Mycobacterium tuberculosis* and *Mycobacterium avium* there is an increased mobilization of mature peripheral T cells recirculating back into this organ, which seem to be related to controlling the thymic infection (De Meis and Savino, 2013; Nobrega et al., 2013). As we reported previously, the thymus is a direct target of *L. infantum* infection (Losada-Barragan et al., 2017) and therefore, according to the proteomic profile, it seems plausible that during a protein restricted diet, the mobilization of peripheral T cells back to the thymus could be diminished, affecting the control of the local infection (Losada-Barragan et al., 2017). In contrast, well-nourished infected animals exhibited a down-regulation of ECHS1 and ACAA2, suggesting a diminished rate of FAO and a proliferative profile in response to infection. However, further assays are needed to clarify this issue.

Additionally, in LPi mice we observed reduced abundance of proteins associated with differentiation processes, such as S100 calcium binding protein A11 (S100a11), endothelial differentiation-related factor 1 (EDF1) and transgelin-2 (TAGLN2). S100a11 has been implicated in the regulation of epidermal (Olsen et al., 1995), chondrocyte (Cecil and Terkeltaub, 2008) and keratinocyte differentiation (He et al., 2009). Furthermore, this protein is able to function as an unconventional inflammatory mediator of altered chondrocyte differentiation and matrix remodeling (Cecil and Terkeltaub, 2008). In cancer cells decreased levels of S100a11 have been associated with invasive forms (Ji et al., 2014; Zhang et al., 2015). EDF1 is a calmodulin binding protein that regulates calmodulin-dependent enzymes involved in the repression of endothelial cell differentiation (Mariotti et al., 2000). Moreover EDF1 is upregulated during early adipogenesis, since silencing of EDF1 in 3T3-L1 cells blocked adipose conversion, showing that its expression is required for the progression of the adipogenic program (Lopez-Victorio et al., 2013). TAGLN and TAGLN2 primarily participate in processes associated with a remodeling of actin cytoskeleton and their role in differentiation has been mainly described in tumor cells. However, the functional meaning of these proteins in the thymus remains to be defined. We presume that downregulation of TAGLN2 and upregulation of TAGLN levels together with downregulation of S100a11 and EDF1 in the IF of LPi mice could be also associated with defective differentiation processes in the thymus.

In general, malnutrition affected the thymus gland in such a way that when the animal is infected, it fails to respond properly to the infection due to all the defects caused by the previous malnutrition. Despite considering that malnutrition is the main detrimental factor for the thymus and therefore has very deleterious consequences in the immune response mediated by cells, we cannot rule out that the infection *per se* is also very harmful to the organ, as has been observed in other infections such as with *Trypanosoma cruzi* (Perez et al., 2012; Gonzalez et al., 2016). However, in this work we do not focus our attention on infection *per se*, because the course of infection is too short to draw conclusions about it. Instead, we call attention to the plethora of alterations that malnutrition induced in the thymic microenvironment and how those defects may impact the response to infection.

Characterization of thymus microarchitecture and thymic secreted proteins in malnourished animals infected with *L. infantum* allowed the identification of new elements in the thymus physiology under such conditions. We demonstrated that the abundance of thymic secreted proteins is altered by protein malnutrition in mice infected with *L. infantum*, likely affecting thymic intercellular communication and basic processes in the thymus. Together, the reduced cortical area, the increased abundance of OxPhos- and β-oxidation-related proteins and the decreased abundance of galectin-1, and Rho GDIa and GDIb, among others, may play a critical role in intrathymic proliferation, dysfunctional thymocyte differentiation and selection and defective T cell migration, all of which can contribute to exacerbate thymic atrophy observed in malnourished mice infected with *L. infantum*. In addition, these elements suggest that a protein-restricted diet modifies both structural and soluble thymic factors, resulting in a non-proliferative microenvironment in *L. infantum* infected mice that could affect the proper mobilization of peripheral T cells into the thymus and toward periphery, affecting the control of the local and systemic infection.

## Data Availability

The datasets generated and analyzed for this study can be found in the Pride Archive https://www.ebi.ac.uk/pride/archive/ with the dataset identifier PXD010414.

## Ethics Statement

This study was carried out in accordance with the recommendations of the Guide for the Care and Use of Laboratory Animals of the National Institutes of Health—Eighth Edition. The protocol was approved by the Instituto Oswaldo Cruz committee for Animal Care and Use (License #LW-27/14). The *L. infantum* strain MCAN/BR/2000/CNV-FEROZ used in this study was provided by the Collection of Leishmania of the Instituto Oswaldo Cruz, Rio de Janeiro (Coleção de Leishmania do Instituto Oswaldo Cruz, CLIOC; http://clioc.fiocruz.br/). This collection is registered in the World Federation for Culture Collections (WFCC-WDCM 731) and is recognized as a Depository Authority by the Brazilian Ministry of the Environment (D.O.U. 05.04.2005).

## Author Contributions

Conceived and designed the experiments: PC, ML-B, AU-P, SC-E, GP. Performed the experiments: ML-B, JD, AR-V, AU-P, SC-E, FR-G, LB, FM, PA, PC. Analyzed the data: ML-B, AU-P, FR-G, RP, DM, PCC, WS, MS-G, GP, PC. Contributed reagents, materials, and analysis tools: AU-P, SC-E, RP, DM, WS, MS-G, PC. Contributed to the writing of the manuscript: ML-B, AU-P, FM, WS, GP, PC. All authors gave final approval of the version to be submitted to publication and agreed to be accountable for all aspects of the work in ensuring that questions related to the accuracy or integrity of any part of the article are appropriately investigated and resolved.

### Conflict of Interest Statement

The authors declare that the research was conducted in the absence of any commercial or financial relationships that could be construed as a potential conflict of interest.
