# Supplementary material for: Thymic Microenvironment Is Modified by Malnutrition and Leishmania infantum Infection
Source: Front Cell Infect Microbiol. 2019 Jul 12;9:252. doi: 10.3389/fcimb.2019.00252 (PMC6639785; doi:10.3389/fcimb.2019.00252)
Supplement: Supplementary file 1 [file Table_1.pdf]

Supplementary Table 1. Identified proteins in thymic IF. Sa Non-classic secretion. Sp. Signal peptide of secretion.

Accesion Number	Unique peptides	Coverage	% Coverage	Length	MolWt (MH)	Sequence Count	Spectrum Count	Name	Gene Name	Secreted	Exosome	Cellular Component
Q921I1	69	0.59	59	697	76655.7	69	522	Serotransferrin	*Tf*	Sp/a	Y	vesicle
Q91X72	28	0.54	54	460	51267.2	28	163	Hemopexin	*Hpx*	Sp/a	Y	vesicle
P07759	23	0.49	49	418	46832	25	162	Serine protease inhibitor A3K	*Serpina3k*	Sp/a	-	membrane-bounded organelle
P27773	16	0.28	28	505	56624.7	16	64	Protein disulfide-isomerase A3	*Pdia3*	Sp	Y	membrane-enclosed lumen
P21614	15	0.26	26	476	53547	15	72	Vitamin D-binding protein	*Gc*	Sp/a	Y	vesicle
Q99KI0	15	0.19	19	780	85392	15	52	Aconitate hydratase, mitochondrial	*Aco2*	-	Y	membrane-bounded organelle
Q01853	14	0.2	20	806	89247.7	14	74	Transitional endoplasmic reticulum ATPase	*Vcp*	-	Y	extracellular organelle
P48036	14	0.41	41	319	35712.2	14	112	Annexin A5	*Anxa5*	-	Y	blood microparticle
Q64727	14	0.15	15	1066	116626.3	14	42	Vinculin	*Vcl*	-	Y	cell junction
P63038	14	0.25	25	573	60899.4	14	80	60 kDa heat shock protein, mitochondrial	*Hspd1*	-	Y	membrane-enclosed lumen
Q9DCW4	14	0.49	49	255	27588	14	88	Electron transfer flavoprotein subunit beta	*Etfb*	-	Y	vesicle
Q61171	14	0.48	48	198	21747	14	104	Peroxiredoxin-2	*Prdx2*	Sa	Y	membrane-bounded organelle
P52480	13	0.27	27	531	57790	13	57	Pyruvate kinase PKM	*Pkm*	-	Y	extracellular matrix
P07724	12	0.83	83	608	68629.7	125	1057	Serum albumin	*Alb*	Sp/a	Y	extracellular matrix
P23953	12	0.25	25	554	60999	19	121	Carboxylesterase 1C	*Ces1c*	Sp/a	Y	membrane-enclosed lumen
P63101	12	0.49	49	245	27735.7	16	110	14-3-3 protein zeta/delta	*Ywhaz*	-	Y	synapse part
E9PZF0	12	0.33	33	267	30162.7	12	77	Nucleoside diphosphate kinase	*Gm20390*	-	-	membrane-bounded organelle
P16015	12	0.46	46	260	29329.6	12	90	Carbonic anhydrase 3	*Ca3*	-	-	Intracellular
P08249	12	0.44	44	338	35570.7	12	51	Malate dehydrogenase,	*Mdh2*	-	Y	membrane-enclosed lumen
P17751	11	0.35	35	299	32153.3	11	69	Triosephosphate isomerase	*Tpi1*	-	Y	vesicle
P14152	11	0.29	29	334	36470.1	11	68	Malate dehydrogenase, cytoplasmic	*Mdh1*	Sa	Y	non-membrane-bounded organelle
Q61599	11	0.55	55	200	22818.5	11	78	Rho GDP-dissociation inhibitor 2	*Arhgdib*	-	Y	vesicle
E9Q616	11	0.03	3	5656	603847.9	11	43	Protein Ahnak	*Ahnak*	-	Y	plasma membrane
O08553	10	0.29	29	572	62220.6	13	51	Dihydropyrimidinase-related	*Dpysl2*	-	Y	site of polarized growth
P11679	10	0.26	26	490	54513.4	13	89	Keratin, type II cytoskeletal 8	*Krt8*	-	Y	plasma membrane
P05784	10	0.36	36	423	47491.2	13	81	Keratin, type I cytoskeletal 18	*Krt18*	Sa	Y	non-membrane-bounded organelle
O35381	10	0.34	34	247	28502.2	12	56	Acidic leucine-rich nuclear phosphoprotein 32 family	*Anp32a*	-	Y	membrane-bounded organelle
Q6IRU2	10	0.4	40	248	28432.4	11	44	Tropomyosin alpha-4 chain	*Tpm4*	-	Y	non-membrane-bounded organelle
P05201	10	0.26	26	413	46200.5	10	27	Aspartate aminotransferase, cytoplasmic	*Got1*	-	Y	cell projection
P99027	9	0.77	77	115	11625.8	9	53	60S acidic ribosomal protein P2	*Rplp2*	-	Y	cell junction
P28665	9	0.05	5	1476	165174.5	9	37	Murinoglobulin-1	*Mug1*	Sp/a	Y	extracellular space
P00920	9	0.34	34	260	28996.5	9	43	Carbonic anhydrase 2	*Ca2*	Sa	Y	neuron part
O35685	8	0.14	14	332	38316.3	8	21	Nuclear migration protein nudC	*Nudc*	-	Y	macromolecular complex
O88569	8	0.22	22	353	37361.7	8	61	Heterogeneous nuclear ribonucleoproteins A2/B1	*H-npa2b1*	-	Y	macromolecular complex
Q99LC5	8	0.23	23	333	34969.5	8	30	Electron transfer flavoprotein subunit alpha, mitochondrial	*Etfa*	Sa	Y	vesicle
P51885	8	0.25	25	338	38222.6	8	40	Lumican	*Lum*	Sp/a	Y	collagen trimer
Q9WVA4	8	0.4	40	199	22363.1	8	41	Transgelin-2	*Tagln2*	Sa	Y	vesicle
Q3U0V1	8	0.13	13	748	76709.7	8	33	Far upstream element-binding protein 2	*Khsrp*	-	Y	non-membrane-bounded organelle
P97807	8	0.14	14	507	54304.1	8	37	Fumarate hydratase,	*Fh*	Sa	Y	vesicle
O70251	7	0.47	47	225	24660.2	8	50	Elongation factor 1-beta	*Eef1b*	Sa	-	macromolecular complex
P56480	7	0.15	15	529	56247.5	7	23	ATP synthase subunit beta, mitochondrial	*Atp5b*	Sa	Y	membrane
P26645	7	0.36	36	309	29625.8	7	68	Myristoylated alanine-rich C-kinase substrate	*Marcks*	-	Y	non-membrane-bounded organelle
Q6ZWZ6	7	0.35	35	132	14487.5	7	39	40S ribosomal protein S12	*Rps12*	Sa	Y	macromolecular complex
Q8BWT1	7	0.22	22	397	41785.4	7	31	3-ketoacyl-CoA thiolase, mitochondrial	*Acaa2*	Sa	Y	membrane
P10639	7	0.48	48	105	11649.6	7	57	Thioredoxin	*Txn*	Sa	Y	vesicle
P40142	7	0.12	12	623	67569.6	7	25	Transketolase	*Tkt*	-	Y	membrane-bounded organelle
P56391	6	0.62	62	86	10046.9	6	37	Cytochrome c oxidase subunit	*Cox6b1*	Sa	-	membrane-enclosed lumen
P08228	6	0.39	39	154	15914.8	6	67	Superoxide dismutase [Cu-Zn]	*Sod1*	Sa	Y	cell body
Q9CQ43	6	0.46	46	162	17355.7	6	42	Deoxyuridine triphosphatase	*Dut*	Sa	Y	extracellular organelle
P20108	6	0.24	24	257	28091.4	6	15	Thioredoxin-dependent peroxide reductase,	*Prdx3*	Sa	Y	vesicle
P62869	6	0.43	43	118	13143.6	6	22	Transcription elongation factor B polypeptide 2	*Tceb2*	Sa	Y	vesicle
P09411	6	0.19	19	417	44504	6	44	Phosphoglycerate kinase 1	*Pgk1*	-	Y	Intracellular
Q99PT1	6	0.29	29	204	23374.8	6	30	Rho GDP-dissociation inhibitor 1	*Arhgdia*	-	Y	plasma membrane
P27546	6	0.08	8	1125	117339	6	15	Microtubule-associated protein	*Map4*	-	Y	plasma membrane
Q62426	6	0.41	41	98	11020.5	6	36	Cystatin-B	*Cstb*	Sa	Y	non-membrane-bounded organelle
Q99KC8	6	0.08	8	793	87069.4	6	18	von Willebrand factor A domain-containing protein 5A	*Vwa5a*	Sa	-	Not assigned
P19157	6	0.17	17	210	23576.1	6	31	Glutathione S-transferase P 1	*Gstp1*	-	Y	membrane

Q9D8B3	6	0.18	18	224	24902.5	6	14	Charged multivesicular body protein 4b	*Chmp4b*	-	Y	plasma membrane
Q9WTP6	6	0.33	33	239	26433.7	6	26	Adenylate kinase 2,	*Ak2*	Sa	Y	cilium
P30416	6	0.19	19	458	51521.9	6	12	Peptidyl-prolyl cis-trans isomerase FKBP4	*Fkbp4*	-	Y	site of polarized growth
Q9D2G2	6	0.11	11	454	48945.5	6	24	2-oxoglutarate dehydrogenase complex component E2	*Dlst*	Sa	Y	oxidoreductase complex
Q8BH95	6	0.23	23	290	31436.2	6	31	Enoyl-CoA hydratase,	*Echs1*	Sa	Y	membrane-enclosed lumen
P01942	5	0.91	91	142	15057.8	58	431	Hemoglobin subunit alpha	*Hba*	-	-	macromolecular complex
P60335	5	0.26	26	356	37455.9	7	41	Poly(rC)-binding protein 1	*Pcbp1*	Sa	Y	macromolecular complex
P01027	5	0.04	4	1663	186347.6	5	9	Complement C3	*C3*	Sp/a	Y	extracellular organelle
Q9CQM5	5	0.37	37	123	13987.7	5	31	Thioredoxin domain-containing protein 17	*Txndc17*	-	Y	vesicle
Q91VW3	5	0.43	43	93	10452.3	5	39	SH3 domain-binding glutamic acid-rich-like protein 3	*Sh3bgrl3*	Sa	Y	cell projection
Q99JY0	5	0.1	10	475	51335.4	5	9	Trifunctional enzyme subunit beta, mitochondrial	*Hadhb*	Sa	Y	nucleoid
Q3THE6	5	0.43	43	183	20664.4	5	25	Ferritin		Sa	Y	Not assigned
Q9R0Q7	5	0.26	26	160	18691.4	5	29	Prostaglandin E synthase 3	*Ptges3*	Sa	Y	vesicle
O08997	5	0.59	59	68	7315.6	5	32	Copper transport protein ATOX1	*Atox1*	Sa	-	Not assigned
Q8C845	5	0.27	27	240	26765.6	5	23	EF-hand domain-containing protein D2	*Efhd2*	-	Y	Not assigned
Q9D1A2	5	0.12	12	475	52715.6	5	15	Cytosolic non-specific dipeptidase	*Cndp2*	-	Y	membrane-bounded organelle
P06151	4	0.27	27	332	36457.2	12	77	L-lactate dehydrogenase A chain	*Ldha*	Sa	Y	cell projection
P04247	4	0.28	28	154	17041	9	20	Myoglobin	*Mb*	Sa	-	membrane bounded organelle
Q93092	4	0.12	12	337	37345.4	4	12	Transaldolase	*Taldo1*	-	Y	extracellular organelle
P34022	4	0.19	19	203	23563.7	4	32	Ran-specific GTPase-activating protein	*Ranbp1*	-	Y	non-membrane-bounded organelle
P50543	4	0.39	39	98	11057.5	4	24	Protein S100-A11	*S100a11*	Sa	Y	cell projection
Q60865	4	0.06	6	707	78102.8	4	12	Caprin-1	*Caprin1*	-	Y	neuron part
Q9CQM9	4	0.14	14	337	37736.4	4	18	Glutaredoxin-3	*Glrx3*	Sa	Y	vesicle
Q9DBP5	4	0.17	17	196	22133.3	4	13	UMP-CMP kinase	*Cmpk1*	Sa	Y	non-membrane-bounded organelle
P16045	4	0.33	33	135	14838.2	4	44	Galectin-1	*Lgals1*	-	Y	extracellular matrix
P37804	4	0.2	20	201	22543.4	4	15	Transgelin	*Tagln*	Sa	Y	Intracellular
P15105	4	0.14	14	373	42074.2	4	8	Glutamine synthetase	*Glul*	-	Y	cell projection
P02088	3	0.95	95	147	15812.1	47	501	Hemoglobin subunit beta-1	*Hbb-b1*	Sa	Y	macromolecular complex
P10126	3	0.37	37	462	50064.1	28	155	Elongation factor 1-alpha 1	*Eef1a1*	-	Y	non-membrane-bounded organelle
P14211	3	0.39	39	416	47946.9	19	154	Calreticulin	*Calr*	-	Y	sperm part
P17182	3	0.37	37	434	47093.2	19	92	Alpha-enolase	*Eno1*	-	Y	membrane
P26040	3	0.15	15	586	69345.6	15	77	Ezrin	*Ezr*	Sa	Y	cell pole
Q60864	3	0.22	22	543	62524.4	13	63	Stress-induced-phosphoprotein 1	*Stip1*	-	Y	membrane-bounded organelle
P68037	3	0.24	24	154	17832.2	6	17	Ubiquitin-conjugating enzyme E2 L3	*Ube2l3*	Sa	Y	membrane-bounded organelle
Q6LD55	3	0.21	21	102	11293.8	3	27	APOAII	*Apoa2*	Sp/a	Y	plasma lipoprotein particle
Q9QXT0	3	0.15	15	182	20736.2	3	9	Protein canopy homolog 2	*Cnpy2*	Sp/a	Y	membrane-bounded organelle
Q9JKX6	3	0.17	17	218	23951	3	6	ADP-sugar pyrophosphatase	*Nudt5*	-	Y	vesicle
Q6P069	3	0.12	12	198	21595.3	3	10	Sorcin	*Sri*	Sa	Y	cell projection
Q99LT0	3	0.45	45	99	11187.8	3	11	Protein dpy-30 homolog	*Dpy30*	Sa	Y	macromolecular complex
Q9D0T1	3	0.09	9	128	14146.5	3	4	NHP2-like protein 1	*Nhp2l1*	-	Y	non-membrane-bounded organelle
P12787	3	0.21	21	146	16073.3	3	7	Cytochrome c oxidase subunit 5A, mitochondrial	*Cox5a*	Sa	Y	membrane
P24472	3	0.12	12	222	25529.4	3	6	Glutathione S-transferase A4	*Gsta4*	-	Y	Intracellular
P99028	3	0.47	47	89	10410	3	24	Cytochrome b-c1 complex subunit 6, mitochondrial	*Uqcrh*	Sa	-	oxidoreductase complex
Q05144	3	0.17	17	192	21409.1	3	16	Ras-related C3 botulinum toxin substrate 2	*Rac2*	Sa	Y	envelope
O89020	3	0.03	3	608	69315.8	3	8	Afamin	*Afm*	Sp/a	Y	vesicle
P62774	3	0.21	21	118	12834.6	3	14	Myotrophin	*Mtpn*	-	Y	neuron part
Q91V76	3	0.09	9	315	34955.3	3	22	Ester hydrolase C11orf54 homolog		Sa	-	vesicle
P97290	3	0.07	7	504	55531.1	3	8	Plasma protease C1 inhibitor	*Serping1*	Sp/a	Y	vesicle
Q6P8J7	3	0.05	5	419	47425.3	3	4	Creatine kinase S-type, mitochondrial	*Ckmt2*	Sa	-	membrane
P63028	3	0.23	23	172	19431.5	3	20	Translationally-controlled tumor protein	*Tpt1*	Sa	Y	vesicle
Q9JL35	3	0.05	5	406	45298.7	3	12	High mobility group nucleosome-binding domain-containing protein 5	*Hmgn5*	-	-	non-membrane-bounded organelle
Q9CQ60	3	0.14	14	257	27219.4	3	5	6-phosphogluconolactonase	*Pgls*	Sa	Y	vesicle
O55042	3	0.25	25	140	14458.2	3	6	Alpha-synuclein	*Snca*	Sa	Y	site of polarized growth
Q66JS7	3	0.17	17	238	26282.7	3	21	Igk protein	*Igk*	Sp/a	Y	Not assigned
Q9CR68	3	0.19	19	274	29331.2	3	12	Cytochrome b-c1 complex subunit Rieske, mitochondrial	*Uqcrfs1*	Sa	Y	oxidoreductase complex
P09528	3	0.23	23	182	21035.2	3	9	Ferritin heavy chain	*Fth1*	Sa	Y	membrane-enclosed lumen
P13634	3	0.15	15	261	28295.2	3	18	Carbonic anhydrase 1	*Ca1*	Sa	Y	membrane-bounded organelle

Q61753	3	0.07	7	533	56531.1	3	5	D-3-phosphoglycerate dehydrogenase	*Phgdh*	Sa	Y	membrane-bounded organelle
P09405	2	0.25	25	707	76658.8	20	155	Nucleolin	*Ncl*	-	Y	non-membrane-bounded organelle
P17742	2	0.54	54	164	17941.8	15	89	Peptidyl-prolyl cis-trans isomerase A	*Ppia*	-	Y	cell junction
P61979	2	0.33	33	463	50926.4	14	83	Heterogeneous nuclear ribonucleoprotein K	*H-npk*	-	Y	cell junction
P99024	2	0.31	31	444	49621	14	96	Tubulin beta-5 chain	*Tubb5*	-	Y	membrane bounded organelle
P13020	2	0.13	13	780	85870.1	11	45	Gelsolin	*Gsn*	Sp/a	Y	cell projection
O35737	2	0.21	21	449	49150.4	10	37	Heterogeneous nuclear ribonucleoprotein H	*H-nph1*	-	Y	macromolecular complex
Q91WJ8	2	0.13	13	651	68479	9	47	Far upstream element-binding protein 1	*Fubp1*	-	Y	membrane-bounded organelle
P62827	2	0.3	30	216	24389.6	9	50	GTP-binding nuclear protein Ran	*Ran*	Sa	Y	Not assigned
P70296	2	0.43	43	187	20799.3	8	55	Phosphatidylethanolamine-binding protein 1	*Pebp1*	-	Y	synapse part
Q99LX0	2	0.29	29	189	19990.4	8	32	Protein DJ-1	*Park7*	Sa	Y	neuron part
Q8QZT1	2	0.18	18	424	44769.3	8	40	Acetyl-CoA acetyltransferase, mitochondrial	*Acat1*	Sa	Y	membrane-enclosed lumen
P31786	2	0.52	52	87	9976.1	8	54	Acyl-CoA-binding protein	*Dbi*	Sa	Y	synapse part
Q9Z0J0	2	0.32	32	149	16413.5	7	27	Epididymal secretory protein E1	*Npc2*	Sp/a	-	membrane bounded organelle
P13707	2	0.12	12	349	37530.4	6	30	Glycerol-3-phosphate dehydrogenase [NAD(+)], cytoplasmic	*Gpd1*	Sa	Y	oxidoreductase complex
Q8R550	2	0.05	5	709	78104.2	5	15	SH3 domain-containing kinase-binding protein 1	*Sh3kbp1*	-	Y	cell junction
Q9QUM9	2	0.16	16	246	27336.8	5	16	Proteasome subunit alpha type-6	*Psma6*	-	Y	non-membrane-bounded organelle
P01869	2	0.06	6	393	43340.7	4	17	Ig gamma-1 chain C region, membrane-bound form	*Ighg1*	-	Y	membrane
Q9WUU7	2	0.07	7	306	33956.2	4	11	Cathepsin Z	*Ctsz*	Sp/a	Y	membrane-bounded organelle
Q9DAW9	2	0.09	9	330	36387.8	4	12	Calponin-3	*Cnn3*	Sa	Y	cell junction
O08749	2	0.04	4	509	54220.2	4	8	Dihydrolipoyl dehydrogenase, mitochondrial	*Dld*	Sa	Y	sperm part
P70195	2	0.07	7	277	29854.2	4	10	Proteasome subunit beta type-7	*Psmb7*	Sa	Y	non-membrane-bounded organelle
P62137	2	0.08	8	330	37497.8	4	7	Serine/threonine-protein phosphatase PP1-alpha catalytic subunit	*Ppp1ca*	Sa	Y	protein serine/threonine phosphatase complex
Q8K354	2	0.12	12	277	30915.9	3	7	Carbonyl reductase [NADPH] 3	*Cbr3*	Sa	Y	membrane-bounded organelle
Q9CXW3	2	0.07	7	229	26475.7	3	6	Calcyclin-binding protein	*Cacybp*	Sa	Y	neuron part
P56375	2	0.21	21	106	11852.1	2	12	Acylphosphatase-2	*Acyp2*	Sa	-	membrane-bounded organelle
Q921M7	2	0.09	9	324	36734.6	2	5	Protein FAM49B	*Fam49b*	-	Y	extracellular organelle
Q91ZX7	2	0.01	1	4545	504392.7	2	2	Prolow-density lipoprotein receptor-related protein 1	*Lrp1*	Sp	Y	cell body
P62830	2	0.16	16	140	14838.1	2	4	60S ribosomal protein L23	*Rpl23*	Sa	Y	cell junction
Q62418	2	0.07	7	436	48651.6	2	9	Drebrin-like protein	*Dbnl*	Sa	Y	cell junction
P35505	2	0.08	8	419	46128	2	10	Fumarylacetoacetase	*Fah*	Sa	Y	vesicle
Q9D6R2	2	0.05	5	366	39595.1	2	5	Isocitrate dehydrogenase [NAD] subunit alpha, mitochondrial	*Idh3a*	-	Y	membrane-bounded organelle
Q9R257	2	0.15	15	190	21035.3	2	4	Heme-binding protein 1	*Hebp1*	-	Y	vesicle
P05125	2	0.18	18	152	16543.4	2	3	Natriuretic peptides A	*Nppa*	Sp/a	-	membrane-bounded organelle
Q9D051	2	0.05	5	359	38894	2	3	Pyruvate dehydrogenase E1 component subunit beta, mitochondrial	*Pdhb*	Sa	Y	oxidoreductase complex
P99026	2	0.03	3	264	29079.3	2	12	Proteasome subunit beta type-4	*Psmb4*	Sa	Y	vesicle
Q8K3C3	2	0.11	11	190	21505.1	2	3	Protein LZIC	*Lzic*	Sa	-	Not assigned
Q9JMG1	2	0.11	11	148	16340.9	2	17	Endothelial differentiation-related factor 1	*Edf1*	Sa	Y	non-membrane-bounded organelle
Q8BKZ9	2	0.02	2	501	53947.2	2	3	Pyruvate dehydrogenase protein X component, mitochondrial	*Pdhx*	Sa	-	membrane-enclosed lumen
O88271	2	0.09	9	295	32883.4	2	2	Craniofacial development protein 1	*Cfdp1*	-	-	extracellular matrix
O70591	2	0.17	17	154	16505.6	2	9	Prefoldin subunit 2	*Pfdn2*	-	Y	non-membrane-bounded organelle
P09542	2	0.18	18	204	22389.3	2	3	Myosin light chain 3	*Myl3*	Sa	Y	macromolecular complex
Q8BMF4	2	0.03	3	642	67880.7	2	3	Pyruvate dehydrogenase complex component E2	*Dlat*	-	Y	oxidoreductase complex
Q91XV3	2	0.12	12	226	22055.6	2	3	Brain acid soluble protein 1	*Basp1*	-	Y	site of polarized growth
Q9EQI5	2	0.11	11	113	12225.5	2	11	Chemokine (C-X-C motif) ligand 7, isoform CRA_b	*Ppbp*	Sp/a	Y	extracellular space
P62311	2	0.14	14	102	11820	2	5	U6 s-NA-associated Sm-like protein LSm3	*Lsm3*	Sa	Y	macromolecular complex

P28667	2	0.14	14	200	20135.4	2	9	MARCKS-related protein	*Marcksl1*	-	Y	membrane
Q9CYG7	2	0.11	11	309	34238.7	2	4	Mitochondrial import receptor subunit TOM34	*Tomm34*	-	Y	membrane
Q9CQQ8	2	0.16	16	103	11610.9	2	8	U6 s-NA-associated Sm-like protein LSm7	*Lsm7*	-	-	macromolecular complex
Q9D6J6	2	0.08	8	248	27249.9	2	4	NADH dehydrogenase [ubiquinone] flavoprotein 2, mitochondrial	*Ndufv2*	Sa	Y	oxidoreductase complex
Q64442	2	0.07	7	357	38206.7	2	3	Sorbitol dehydrogenase	*Sord*	Sa	Y	cilium
Q91XL1	2	0.08	8	342	37389.5	2	12	Leucine-rich HEV glycoprotein	*Lrg1*	Sp/a	Y	vesicle
P97825	2	0.16	16	154	16052.8	2	2	Hematological and neurological expressed 1 protein	*Hn1*	-	Y	membrane-bounded organelle
P32020	2	0.03	3	547	59069.7	2	5	Non-specific lipid-transfer protein	*Scp2*	Sa	-	vesicle
P26262	2	0.04	4	638	71317.9	2	2	Plasma kallikrein	*Klkb1*	Sp/a	Y	membrane-bounded organelle
P52503	2	0.22	22	116	12993.7	2	5	NADH dehydrogenase [ubiquinone] iron-sulfur protein 6, mitochondrial	*Ndufs6*	Sa	-	oxidoreductase complex
Q9D7S9	2	0.15	15	219	24542.3	2	4	Charged multivesicular body protein 5	*Chmp5*	-	Y	membrane
Q61823	2	0.05	5	469	51652	2	6	Programmed cell death protein 4	*Pdcd4*	-	Y	membrane-bounded organelle
Q91WU5	2	0.09	9	376	41749.1	2	4	Arsenite methyltransferase	*As3mt*	-	-	membrane-bounded organelle
Q9R1T2	2	0.09	9	350	38577.6	2	3	SUMO-activating enzyme subunit 1	*Sae1*	Sa	Y	macromolecular complex
P18760	1	0.58	58	166	18529.7	23	159	Cofilin-1	*Cfl1*	Sa	Y	cell projection
P07758	1	0.31	31	413	45955.5	18	119	Alpha-1-antitrypsin 1-1	*Serpina1a*	Sp/a	Y	membrane-bounded organelle
Q00623	1	0.38	38	264	30578.6	16	87	Apolipoprotein A-I	*Apoa1*	Sp/a	Y	plasma lipoprotein particle
P99029	1	0.52	52	210	21865.5	15	82	Peroxiredoxin-5, mitochondrial	*Prdx5*	Sa	Y	membrane-enclosed lumen
P60710	1	0.3	30	375	41691.7	14	86	Actin, cytoplasmic 1	*Actb*	-	Y	extracellular space
P06728	1	0.28	28	395	44983	14	74	Apolipoprotein A-IV	*Apoa4*	Sp	Y	plasma lipoprotein particle
P16858	1	0.38	38	333	35769.2	13	76	Glyceraldehyde-3-phosphate dehydrogenase	*Gapdh*	Sa	Y	Intracellular
P35700	1	0.38	38	199	22144.3	13	99	Peroxiredoxin-1	*Prdx1*	Sa	Y	membrane-enclosed lumen
P05064	1	0.43	43	364	39313.3	13	34	Fructose-bisphosphate aldolase A	*Aldoa*	-	Y	macromolecular complex
P68369	1	0.27	27	451	50085.6	12	92	Tubulin alpha-1A chain	*Tuba1a*	-	Y	Intracellular
P29699	1	0.34	34	345	37283.7	12	55	Alpha-2-HS-glycoprotein	*Ahsg*	Sp/a	Y	membrane-bounded organelle
P63158	1	0.27	27	215	24860.2	11	86	High mobility group protein B1	*Hmgb1*	-	Y	non-membrane-bounded organelle
Q9EQ20	1	0.23	23	535	57860.5	11	38	Methylmalonate-semialdehyde dehydrogenase [acylating], mitochondrial	*Aldh6a1*	Sa	Y	membrane bounded organelle
Q9EQU5	1	0.33	33	289	33339.7	10	65	Protein SET	*Set*	-	Y	macromolecular complex
P17225	1	0.22	22	527	56424.9	9	48	Polypyrimidine tract-binding protein 1	*Ptbp1*	-	Y	membrane-bounded organelle
P18242	1	0.17	17	410	44907	9	49	Cathepsin D	*Ctsd*	Sp/a	Y	membrane-bounded organelle
Q9CPU0	1	0.44	44	184	20778.4	9	41	Lactoylglutathione lyase	*Glo1*	-	Y	membrane-bounded organelle
P63242	1	0.36	36	154	16803.4	9	68	Eukaryotic translation initiation factor 5A-1	*Eif5a*	-	Y	neuron part
P10107	1	0.22	22	346	38692	8	35	Annexin A1	*Anxa1*	-	Y	plasma membrane
P19221	1	0.15	15	618	70205.6	8	19	Prothrombin	*F2*	Sp/a	Y	membrane-bounded organelle
Q61838	1	0.06	6	1495	165729.8	8	35	Alpha-2-macroglobulin	*A2m*	Sp/a	Y	extracellular space
Q9CZX8	1	0.34	34	145	16057.6	7	42	40S ribosomal protein S19	*Rps19*	Sa	Y	cell junction
P97371	1	0.23	23	249	28636.9	7	48	Proteasome activator complex subunit 1	*Psme1*	-	Y	macromolecular complex
Q9R0P5	1	0.39	39	165	18491.5	7	25	Destrin	*Dstn*	Sa	Y	non-membrane-bounded organelle
Q9WUK2	1	0.24	24	248	27306.4	7	34	Eukaryotic translation initiation factor 4H	*Eif4h*	Sa	Y	membrane
P01872	1	0.18	18	454	49922.3	7	22	Ig mu chain C region	*Ighm*	Sa	Y	plasma membrane
P26350	1	0.23	23	111	12229	6	77	Prothymosin alpha	*Ptma*	-	Y	membrane-bounded organelle
P14824	1	0.08	8	673	75819.4	6	17	Annexin A6	*Anxa6*	-	Y	cell junction
Q61937	1	0.2	20	292	32521.8	6	30	Nucleophosmin	*Npm1*	Sa	Y	cell junction
O35215	1	0.57	57	118	13050.8	6	30	D-dopachrome decarboxylase	*Ddt*	-	Y	membrane-bounded organelle
P32261	1	0.1	10	465	51952.6	6	23	Antithrombin-III	*Serpinc1*	Sp/a	Y	membrane-bounded organelle
P97315	1	0.26	26	193	20551.7	6	15	Cysteine and glycine-rich protein 1	*Csrp1*	-	Y	cell junction
P26638	1	0.11	11	512	58333.7	6	23	Serine--tRNA ligase, cytoplasmic	*Sars*	-	Y	membrane-bounded organelle
Q01339	1	0.19	19	345	38574.9	6	24	Beta-2-glycoprotein 1	*Apoh*	Sp/a	Y	plasma lipoprotein particle
Q9QXC1	1	0.13	13	388	42667.4	6	15	Fetuin-B	*Fetub*	Sp/a	-	membrane-bounded organelle

Q60611	1	0.06	6	764	85808.4	6	21	DNA-binding protein SATB1	*Satb1*	-	Y	macromolecular complex
P32067	1	0.12	12	415	47708.9	6	14	Lupus La protein homolog	*Ssb*	-	Y	macromolecular complex
Q8CDN6	1	0.16	16	289	32198.7	5	17	Thioredoxin-like protein 1	*Txnl1*	-	Y	macromolecular complex
O08795	1	0.08	8	521	58737.7	5	19	Glucosidase 2 subunit beta	*Prkcsh*	Sp	Y	membrane-bounded organelle
O08784	1	0.04	4	1320	134902.8	5	8	Treacle protein	*Tcof1*	-	Y	non-membrane-bounded organelle
P08074	1	0.21	21	244	25923.6	5	13	Carbonyl reductase [NADPH] 2	*Cbr2*	Sa	-	membrane-enclosed lumen
P23198	1	0.15	15	183	20824.3	5	29	Chromobox protein homolog 3	*Cbx3*	Sa	Y	envelope
Q921F2	1	0.1	10	414	44501.3	4	12	TAR DNA-binding protein 43	*Tardbp*	-	Y	membrane-bounded organelle
P51125	1	0.05	5	788	84852.6	4	10	Calpastatin	*Cast*	-	-	membrane
Q9CQI6	1	0.2	20	142	15916	4	33	Coactosin-like protein	*Cotl1*	Sa	Y	non-membrane-bounded organelle
Q8VEK3	1	0.04	4	800	87844.7	4	16	Heterogeneous nuclear ribonucleoprotein U	*H-npu*	-	Y	non-membrane-bounded organelle
Q6NZB0	1	0.1	10	253	29776.4	4	21	DnaJ homolog subfamily C member 8	*Dnajc8*	Sa	Y	Not assigned
P20918	1	0.05	5	812	90730.9	4	5	Plasminogen	*Plg*	Sp	Y	plasma membrane
Q64339	1	0.21	21	161	17868.2	4	19	Ubiquitin-like protein ISG15	*Isg15*	Sa	Y	extracellular space
P11352	1	0.17	17	201	22298.2	4	4	Glutathione peroxidase 1	*Gpx1*	Sa	Y	membrane bounded organelle
P62889	1	0.25	25	115	12757.7	4	11	60S ribosomal protein L30	*Rpl30*	-	Y	cell junction
P34884	1	0.33	33	115	12478.2	4	23	Macrophage migration inhibitory factor	*Mif*	Sa	Y	extracellular space
P97352	1	0.34	34	98	11132.9	4	8	Protein S100-A13	*S100a13*	Sa	Y	extracellular space
O35887	1	0.09	9	315	37022.5	3	10	Calumenin	*Calu*	Sp/a	Y	membrane-enclosed lumen
Q9DBD0	1	0.03	3	700	76697.5	3	3	Inhibitor of carbonic anhydrase	*Ica*	Sp/a	-	extracellular space
Q4KML4	1	0.27	27	81	9006.8	3	19	Costars family protein ABRACL	*Abracl*	Sa	-	Not assigned
P46412	1	0.1	10	226	25390.8	3	5	Glutathione peroxidase 3	*Gpx3*	Sp/a	Y	membrane-bounded organelle
Q64213	1	0.02	2	653	70340.1	3	11	Splicing factor 1	*Sf1*	-	-	macromolecular complex
Q9D2M8	1	0.13	13	145	16338.2	3	12	Ubiquitin-conjugating enzyme E2 variant 2	*Ube2v2*	Sa	Y	macromolecular complex
O35900	1	0.32	32	95	10809.6	3	11	U6 s-NA-associated Sm-like protein LSm2	*Lsm2*	Sa	Y	macromolecular complex
P56399	1	0.02	2	858	95754.3	3	12	Ubiquitin carboxyl-terminal hydrolase 5	*Usp5*	-	Y	membrane-bounded organelle
Q9CQE5	1	0.16	16	181	21119.6	3	4	Regulator of G-protein signaling 10	*Rgs10*	Sa	Y	cell projection
Q9WTX5	1	0.15	15	163	18642.2	3	13	S-phase kinase-associated protein 1	*Skp1*	Sa	Y	macromolecular complex
Q64152	1	0.13	13	204	21999.4	3	10	Transcription factor BTF3	*Btf3*	-	Y	Intracellular
P26039	1	0.01	1	2541	269635.1	3	6	Talin-1	*Tln1*	-	Y	cell projection
Q61166	1	0.1	10	268	29979.2	3	14	Microtubule-associated protein RP/EB family member 1	*Mapre1*	-	Y	cell projection
O54724	1	0.07	7	392	43909	3	3	Polymerase I and transcript release factor	*Ptrf*	Sa	Y	plasma membrane
Q9CQR2	1	0.22	22	83	9117.5	3	20	40S ribosomal protein S21	*Rps21*	Sa	Y	macromolecular complex
P28798	1	0.02	2	589	63395.1	2	5	Granulins	*Grn*	Sp/a	Y	membrane-bounded organelle
Q9JMD0	1	0.03	3	495	52740.1	2	3	BUB3-interacting and GLEBS motif-containing protein ZNF207	*Znf207*	-	-	non-membrane-bounded organelle
P06909	1	0.01	1	1234	139028.6	2	3	Complement factor H	*Cfh*	Sp	Y	membrane-bounded organelle
P61961	1	0.18	18	85	9093.9	2	9	Ubiquitin-fold modifier 1	*Ufm1*	-	-	membrane-bounded organelle
A6X935	1	0.01	1	942	104575.9	2	2	Inter alpha-trypsin inhibitor, heavy chain 4	*Itih4*	Sp/a	Y	membrane
Q9D7G0	1	0.04	4	318	34793.9	2	6	Ribose-phosphate pyrophosphokinase 1	*Prps1*	-	Y	Not assigned
P80316	1	0.03	3	541	59567.9	2	3	T-complex protein 1 subunit epsilon	*Cct5*	-	Y	non-membrane-bounded organelle
P63073	1	0.05	5	217	25019.5	2	5	Eukaryotic translation initiation factor 4E	*Eif4e*	Sa	Y	non-membrane-bounded organelle
Q8R5L1	1	0.04	4	279	30987.5	2	12	Complement component 1 Q subcomponent-binding protein, mitochondrial	*C1qbp*	Sa	Y	membrane-enclosed lumen
P47941	1	0.04	4	303	33791	2	8	Crk-like protein	*Crkl*	Sa	Y	Intracellular
Q8K1I7	1	0.02	2	493	50031.8	2	5	WAS/WASL-interacting protein family member 1	*Wipf1*	-	Y	cell projection

Single peptide identified proteins in thymic IF. Sa Non-classic secretion. Sp. Signal peptide of secretion.

Accesion Number	Coverage	Length	Primary Score	DeltCN	M+H+	CalcM+H+	Name	Bayesian Score	Sequence	z	m/z	Mass error	Secreted	Exosomal
Q63918	0.02	418	3.0259	0.2884	1275.755053	1275.746937	Serum deprivation-response protein	0.48675	QINLEGSVK(+144.100000)	2	637.8775265	-0.008116	Sa	Y
			3.3476	0.258	1275.753588	1275.746937		0.47396	QINLEGSVK(+144.100000)	2	637.876794	-0.006651		
Q8VCC1	0.06	269	3.2633	0.38	2033.131321	2033.126848	15-hydroxyprostaglandin dehydrogenase [NAD(+)]	0.56165	GIHFQDYDISPLLVK(+144.100000)	3	677.7104403	-0.004473	-	Y
			2.5022	0.1256	2033.132054	2033.126848		0.40597	GIHFQDYDISPLLVK(+144.100000)	3	677.7106847	-0.005206		
P11031	0.09	127	3.6566	0.3412	1404.706957	1404.706636	Activated RNA polymerase II transcriptional coactivator p15	0.48971	EQISDIDDAVR	2	702.3534785	-0.000321	Sa	Y
			3.2403	0.3084	1404.704882	1404.706636		0.45344	EQISDIDDAVR	2	702.352441	0.001754		
			3.1659	0.3	1404.70598	1404.706636		0.44428	EQISDIDDAVR	2	702.35299	0.000656		
			3.0738	0.2872	1404.711474	1404.706636		0.41894	EQISDIDDAVR	2	702.355737	-0.004838		
			2.9629	0.285	1404.711474	1404.706636		0.41861	EQISDIDDAVR	2	702.355737	-0.004838		
			3.6078	0.2854	1404.711962	1404.706636		0.41491	EQISDIDDAVR	2	702.355981	-0.005326		
			3.5868	0.2706	1404.711229	1404.706636		0.40116	EQISDIDDAVR	2	702.3556145	-0.004593		
			2.7204	0.2562	1404.710131	1404.706636		0.39973	EQISDIDDAVR	2	702.3550655	-0.003495		
			3.3843	0.2523	1404.705492	1404.706636		0.39032	EQISDIDDAVR	2	702.352746	0.001144		
			3.4866	0.2436	1404.70537	1404.706636		0.38631	EQISDIDDAVR	2	702.352685	0.001266		
			2.8979	0.2287	1404.707933	1404.706636		0.37746	EQISDIDDAVR	2	702.3539665	-0.001297		
			2.8481	0.2248	1404.714403	1404.706636		0.35274	EQISDIDDAVR	2	702.3572015	-0.007767		
Q9CPX6	0.04	314	2.6324	0.4607	1734.036425	1734.025009	Ubiquitin-like-conjugating enzyme ATG3	0.57615	ALEVAEYLTPVLK(+144.100000)	2	867.0182125	-0.011416	Sa	Y
Q61425	0.04	314	3.2182	0.2225	1429.896532	1429.887836	Hydroxyacyl-coenzyme A dehydrogenase, mitochondrial	0.29624	LLVPYLIEAVR	2	714.948266	-0.008696	Sa	Y
			3.3547	0.2341	1429.897509	1429.887836		0.28549	LLVPYLIEAVR	2	714.9487545	-0.009673		
			2.2354	0.1136	1429.889452	1429.887836		0.2362	LLVPYLIEAVR	2	714.944726	-0.001616		
Q9CQ45	0.05	171	2.9039	0.2166	1251.673143	1251.668066	Neudesin	0.28982	LFTEEELAR	2	625.8365715	-0.005077	Sp/a	-
P70124	0.02	375	2.7163	0.2502	1205.728441	1205.730224	Serpin B5	0.46228	ASLESLGLK(+144.100000)	2	602.8642205	0.001783	-	Y
			2.7195	0.2761	1205.737963	1205.730224		0.46069	ASLESLGLK(+144.100000)	2	602.8689815	-0.007739		
A2ADY9	0.05	399	3.8304	0.2861	2485.309178	2485.298284	Protein DDI1 homolog 2	0.42167	EDIRPEEIADQELAEAIQK(+144.100000)	3	828.4363927	-0.010894	Sa	Y
Q3TA75	0.02	655	2.5209	0.2343	1813.941624	1810.932961	Putative uncharacterized protein (Fragment)	0.39249	DGERPGPGPLLVGCSR	3	604.647208	-3.008663	-	Y
P10493	0.01	1245	2.8045	0.3582	1207.741381	1207.740001	Nidogen-1	0.57926	GLVGFLWK(+144.100000)	2	603.8706905	-0.00138	Sp/a	Y
			2.5918	0.3716	1207.742601	1207.740001		0.57737	GLVGFLWK(+144.100000)	2	603.8713005	-0.0026		
Q8VDM6	0.01	859	2.5663	0.4035	1661.908861	1661.89809	Heterogeneous nuclear ribonucleoprotein U-like protein 1	0.55252	AIVICPTDEDLK(+144.100000)	2	830.9544305	-0.010771	-	y
			1.9167	0.2661	1661.902758	1661.89809		0.42747	AIVICPTDEDLK(+144.100000)	2	830.951379	-0.004668		
P57759	0.03	262	2.5861	0.2508	1178.704271	1178.699306	Endoplasmic reticulum resident protein 29	0.36239	SLNILTAFR	2	589.3521355	-0.004965	Sp/a	Y
			2.8269	0.2264	1178.704149	1178.699306		0.34981	SLNILTAFR	2	589.3520745	-0.004843		
			2.8041	0.2136	1178.704393	1178.699306		0.33643	SLNILTAFR	2	589.3521965	-0.005087		
			2.8211	0.226	1178.705492	1178.699306		0.33596	SLNILTAFR	2	589.352746	-0.006186		
			2.7527	0.1967	1178.699755	1178.699306		0.33462	SLNILTAFR	2	589.3498775	-0.000449		
			2.8967	0.173	1178.701464	1178.699306		0.32731	SLNILTAFR	2	589.350732	-0.002158		
			2.544	0.1959	1178.703783	1178.699306		0.32074	SLNILTAFR	2	589.3518915	-0.004477		
			2.8301	0.1773	1178.698656	1178.699306		0.31908	SLNILTAFR	2	589.349328	0.00065		
			2.576	0.1479	1178.699022	1178.699306		0.30358	SLNILTAFR	2	589.349511	0.000284		
			2.4674	0.1505	1178.705492	1178.699306		0.29963	SLNILTAFR	2	589.352746	-0.006186		
Q60692	0.05	238	2.9008	0.231	1316.727953	1316.726978	Proteasome subunit beta type-6	0.31506	LAAIQESGVER	2	658.3639765	-0.000975	Sa	Y
P38647	0.02	679	3.0845	0.2707	1386.783495	1386.780076	Stress-70 protein, mitochondrial	0.34218	DAGQISGLNVLR	2	693.3917475	-0.003419	-	Y
Q9CQ75	0.14	99	4.1352	0.4324	1662.873339	1662.875827	NADH dehydrogenase [ubiquinone] 1 alpha subcomplex subunit 2	0.60882	TVSLNNLSADEVTR	2	831.4366695	0.002488	Sa	-
			2.3113	0.2	1662.875902	1662.875827		0.28119	TVSLNNLSADEVTR	2	831.437951	-7.5E-05		
			1.9605	0.0949	1662.886278	1662.875827		0.24365	TVSLNNLSADEVTR	2	831.443139	-0.010451		
Q8BH93	0.08	242	3.3217	0.1908	2357.299778	2357.297447	MAPK-interacting and spindle-stabilizing protein-like	0.1614	ADALPEQSSAK(+144.100000)PPAVTNTK(+144.100000)	3	785.7665927	-0.002331	Sa	Y
Q64726	0.05	307	3.0659	0.5034	1995.119921	1995.111198	Zinc-alpha-2-glycoprotein	0.77153	EIPAWIPLDPAAANTK(+144.100000)	2	997.5599605	-0.008723	Sp/a	Y
			2.4111	0.4683	1995.114428	1995.111198		0.69332	EIPAWIPLDPAAANTK(+144.100000)	2	997.557214	-0.00323		
			2.574	0.4227	1995.116015	1995.111198		0.6459	EIPAWIPLDPAAANTK(+144.100000)	2	997.5580075	-0.004817		
			2.1599	0.4009	1995.115282	1995.111198		0.59486	EIPAWIPLDPAAANTK(+144.100000)	2	997.557641	-0.004084		
			2.1573	0.4032	1995.119921	1995.111198		0.58356	EIPAWIPLDPAAANTK(+144.100000)	2	997.5599605	-0.008723		
			2.2869	0.299	1995.116381	1995.111198		0.51785	EIPAWIPLDPAAANTK(+144.100000)	2	997.5581905	-0.005183		
Q9D817	0.04	178	2.742	0.3141	1089.654223	1089.655651	Putative uncharacterized protein	0.54473	AVFWKVVP	2	544.8271115	0.001428	-	-
			3.0901	0.2692	1089.655932	1089.655651		0.51318	AVFWKVVP	2	544.827966	-0.000281		
			1.9417	0.2764	1089.6541	1089.655651		0.46835	AVFWKVVP	2	544.82705	0.001551		
Q8BHA3	0.13	168	2.8913	0.3734	2428.41135	2428.402343	Probable D-tyrosyl-tRNA(Tyr) deacylase 2	0.45897	HVSILDLPGDVLIIPQATLGGR	3	809.47045	-0.009007	Sa	-
Q9ESB3	0.03	525	3.2851	0.3099	2361.219151	2361.217514	Histidine-rich glycoprotein	0.48049	DSPVLLDFFEDSELYRK(+144.100000)	3	787.0730503	-0.001637	Sp	Y

Q9JHU9	0.02	557	3.2324	0.411	1635.996996	1635.98823	Inositol-3-phosphate synthase 1	0.61351	SVLVDFLIGSGLK(+144.100000)	2	817.998498	-0.008766	Sa	Y
			1.9809	0.2982	1635.99309	1635.98823		0.46748	SVLVDFLIGSGLK(+144.100000)	2	817.996545	-0.00486		
			1.6312	0.3503	1635.997728	1635.98823		0.44149	SVLVDFLIGSGLK(+144.100000)	2	817.998864	-0.009498		
Q9D8S9	0.09	137	3.479	0.2908	1640.936937	1640.931885	BolA-like protein 1	0.40486	LEQALSPEVLELR	2	820.4684685	-0.005052	Sa	-
			4.2064	0.2699	1640.93828	1640.931885		0.37958	LEQALSPEVLELR	2	820.46914	-0.006395		
			3.7392	0.2554	1640.933519	1640.931885		0.37668	LEQALSPEVLELR	2	820.4667595	-0.001634		
			3.5935	0.2502	1640.938891	1640.931885		0.36273	LEQALSPEVLELR	2	820.4694455	-0.007006		
			3.9466	0.2335	1640.937426	1640.931885		0.3514	LEQALSPEVLELR	2	820.468713	-0.005541		
			2.897	0.1396	1640.938036	1640.931885		0.28093	LEQALSPEVLELR	2	820.469018	-0.006151		
P62077	0.13	83	2.7715	0.1402	1351.770311	1351.768114	Mitochondrial import inner membrane translocase subunit Tim8 B	0.23357	FIDTTLAITGR	2	675.8851555	-0.002197	Sa	-
Q8BK67	0.02	520	2.9721	0.2213	1549.918017	1549.916176	Protein RCC2	0.2879	DGQILPVPNVVVR	2	774.9590085	-0.001841	-	Y
O35127	0.14	126	3.7057	0.5101	2057.157274	2057.149089	Protein C10	0.73889	VVLAEVIQAFSAPENAVR	2	1028.578637	-0.008185	Sa	-
Q9D1P4	0.05	331	2.6299	0.2551	2280.34531	2280.337796	Cysteine and histidine-rich domain-containing protein 1	0.40319	FQEHIIQAPK(+144.100000)PVEAIK(+144.100000)	3	760.1151033	-0.007514	Sa	Y
O70209	0.05	316	2.7566	0.3589	1774.000414	1773.995883	PDZ and LIM domain protein 3	0.46465	LSGGIDFNQPLVITR	2	887.000207	-0.004531	Sa	-
			2.5851	0.3058	1773.996996	1773.995883		0.38934	LSGGIDFNQPLVITR	2	886.998498	-0.001113		
			2.7452	0.2799	1774.000292	1773.995883		0.3674	LSGGIDFNQPLVITR	2	887.000146	-0.004409		

Differentially abundant proteins in the IF of the thymus of well-nourished mice infected with L. infantum BALB/c mice (CPi).

ProtID	Daltons	Sequence Count	Spectral Count	Avg Log Fold	Fold change	Stouffers P Value	Description	Gene	up/down
Q921I1	76655.71	26	179	1.567	4.8	0.000	Serotransferrin	Tf	down
Q9DCW4	27587.97	9	61	1.54	4.7	0.000	Electron transfer flavoprotein subunit beta	Etfb	down
P07759	46831.98	6	32	1.838	6.3	0.050	Serine protease inhibitor A3K	Serpina3k	down
P05201	46200.5	5	20	0.922	2.5	0.000	Aspartate aminotransferase, cytoplasmic	Got1	down
Q91X72	51267.17	5	18	0.908	2.5	0.001	Hemopexin	Hpx	down
P16015	29329.63	4	45	0.685	2.0	0.001	Carbonic anhydrase 3	Ca3	down
P97807	54304.06	4	28	0.65	1.9	0.002	Fumarate hydratase, mitochondrial	Fh	down
Q62426	11020.53	4	25	2.004	7.4	0.003	Cystatin-B	Cstb	down
P07724	68629.7	4	11	0.758	2.1	0.042	Serum albumin	Alb	down
P56391	10046.87	3	24	0.545	1.7	0.004	Cytochrome c oxidase subunit 6B1	Cox6b1	down
P19157	23576.1	3	22	0.747	2.1	0.006	Glutathione S-transferase P 1	Gstp1	down
Q8BH95	31436.2	3	19	0.839	2.3	0.004	Enoyl-CoA hydratase, mitochondrial	Echs1	down
Q8BWT1	41785.43	2	20	0.86	2.4	0.013	3-ketoacyl-CoA thiolase, mitochondrial	Acaa2	down
P17751	32153.25	2	16	0.958	2.6	0.008	Triosephosphate isomerase	Tpi1	down
P21614	53546.98	2	16	1.904	6.7	0.006	Vitamin D-binding protein	Gc	down
O88569	37361.71	2	15	0.504	1.7	0.013	Heterogeneous nuclear ribonucleoproteins A2/B1	Hnrnpa2b1	down
P56480	56247.46	2	12	1.861	6.4	0.007	ATP synthase subunit beta, mitochondrial	Atp5b	down
P14152	36470.07	2	12	1.293	3.6	0.008	Malate dehydrogenase, cytoplasmic	Mdh1	down
Q64727	116626.3	2	11	0.801	2.2	0.008	Vinculin	Vcl	down
P35505	46128.03	2	10	0.683	2.0	0.010	Fumarylacetoacetase	Fah	down
P20108	28091.4	2	8	1.029	2.8	0.007	Thioredoxin-dependent peroxide reductase, mitochondrial	Prdx3	down
P24472	25529.38	2	5	1.604	5.0	0.009	Glutathione S-transferase A4	Gsta4	down
O35381	28502.23	1	12	0.685	2.0	0.011	Acidic leucine-rich nuclear phosphoprotein 32 family member A	Anp32a	down
P09411	44503.98	1	10	0.571	1.8	0.034	Phosphoglycerate kinase 1	Pgk1	down
P27773	56624.67	1	10	1.181	3.3	0.024	Protein disulfide-isomerase A3	Pdia3	down
Q3THE6	20664.39	1	10	0.485	1.6	0.010	Ferritin	2 SV=1	down
P63038	60899.38	1	10	0.604	1.8	0.010	60 kDa heat shock protein, mitochondrial	Hspd1	down
Q91V76	34955.33	1	9	1.201	3.3	0.010	Ester hydrolase C11orf54 homolog	2 SV=1	down
P63101	27735.73	1	8	0.848	2.3	0.025	14-3-3 protein zeta/delta	Ywhaz	down
E9PZF0	30162.67	1	6	1.495	4.5	0.010	Nucleoside diphosphate kinase	Gm20390	down
P56375	11852.05	1	5	0.739	2.1	0.018	Acylphosphatase-2	Acyp2	down
P12787	16073.27	1	5	1.061	2.9	0.015	Cytochrome c oxidase subunit 5A, mitochondrial	Cox5a	down
P08249	35570.75	1	5	0.778	2.2	0.010	Malate dehydrogenase, mitochondrial	Mdh2	down
Q9D2G2	48945.47	1	5	1.401	4.1	0.018	2-oxoglutarate dehydrogenase complex component E2	Dlst	down
Q99LC5	34969.49	1	4	0.849	2.3	0.010	Electron transfer flavoprotein subunit alpha, mitochondrial	Etfa	down
P51885	38222.6	1	4	1.653	5.2	0.010	Lumican	Lum	down
P15105	42074.25	1	4	0.43	1.5	0.010	Glutamine synthetase	Glul	down
P60335	37455.93	1	3	2.517	12.4	0.010	Poly(rC)-binding protein 1	Pcbp1	down
Q01853	89247.71	1	2	3.127	22.8	0.010	Transitional endoplasmic reticulum ATPase	Vcp	down
P62869	13143.64	1	2	1.583	4.9	0.034	Transcription elongation factor B polypeptide 2	Tceb2	down
Q99LT0	11187.76	1	2	1.533	4.6	0.010	Protein dpy-30 homolog	Dpy30	down
Q6P8J7	47425.31	1	2	0.458	1.6	0.044	Creatine kinase S-type, mitochondrial	Ckmt2	down
P48036	35712.2	1	2	2.241	9.4	0.042	Annexin A5	Anxa5	down
P32020	59069.74	1	2	1.67	5.3	0.017	Non-specific lipid-transfer protein	Scp2	down
O08553	62220.58	1	2	0.476	1.6	0.010	Dihydropyrimidinase-related protein 2	Dpysl2	down
Q61937	32521.8	1	2	0.539	1.7	0.010	Nucleophosmin	Npm1	down
P70195	29854.24	1	2	3.829	46.0	0.010	Proteasome subunit beta type-7	Psmb7	down
P27546	117338.98	1	7	-0.421	1.5	0.011	Microtubule-associated protein 4	Map4	up
Q9CQM5	13987.73	1	6	-0.698	2.0	0.010	Thioredoxin domain-containing protein 17	Txndc17	up
Q9D1A2	52715.6	1	4	-0.551	1.7	0.025	Cytosolic non-specific dipeptidase	Cndp2	up
Q61823	51651.98	1	3	-0.48	1.6	0.034	Programmed cell death protein 4	Pdcd4	up
P10126	50064.09	1	3	-0.626	1.9	0.010	Elongation factor 1-alpha 1	Eef1a1	up
P63073	25019.48	1	3	-0.469	1.6	0.017	Eukaryotic translation initiation factor 4E	Eif4e	up
P26350	12229.04	1	2	-3.136	23.0	0.017	Prothymosin alpha	Ptma	up
P60710	41691.72	1	2	-0.416	1.5	0.028	Actin, cytoplasmic 1	Actb	up

Differentially abundant proteins in the IF of the thymus of protein malnourished BALB/c mice (LP).

ProtID	Daltons	Sequence Count	Spectral Count	Avg Log Fold	Fold change	Stouffers P Value	Description	Gene	up/down
Q921I1	76655.71	36	282	1.01	2.7	0.000	Serotransferrin	Tf	down
P07759	46831.98	15	129	2.068	7.9	0.000	Serine protease inhibitor A3K	Serpina3k	down
P48036	35712.2	9	93	0.814	2.3	0.000	Annexin A5	Anxa5	down
P16015	29329.63	9	85	1.047	2.8	0.000	Carbonic anhydrase 3	Ca3	down
Q91X72	51267.17	11	76	0.805	2.2	0.000	Hemopexin	Hpx	down
Q01853	89247.71	12	68	1.436	4.2	0.000	Transitional endoplasmic reticulum ATPase	Vcp	down
P26645	29625.81	7	68	0.459	1.6	0.000	Myristoylated alanine-rich C-kinase substrate	Marcks	down
P11679	54513.45	6	59	1.642	5.2	0.002	Keratin, type II cytoskeletal 8	Krt8	down
E9PZF0	30162.67	7	57	1.505	4.5	0.000	Nucleoside diphosphate kinase	Gm20390	down
P52480	57789.99	8	50	0.942	2.6	0.000	Pyruvate kinase PKM	Pkm	down
Q61171	21747.05	6	50	0.996	2.7	0.005	Peroxiredoxin-2	Prdx2	down
P05784	47491.21	6	43	1.713	5.5	0.000	Keratin, type I cytoskeletal 18	Krt18	down
P63101	27735.73	5	42	1.322	3.8	0.000	14-3-3 protein zeta/delta	Ywhaz	down
P16045	14838.19	3	39	0.569	1.8	0.003	Galectin-1	Lgals1	down
P99027	11625.82	4	38	1.595	4.9	0.001	60S acidic ribosomal protein P2	Rplp2	down
Q6ZWZ6	14487.47	4	34	0.67	2.0	0.001	40S ribosomal protein S12	Rps12	down
Q61599	22818.48	4	34	1.561	4.8	0.001	Rho GDP-dissociation inhibitor 2	Arhgdib	down
P28665	165174.53	5	32	1.323	3.8	0.001	Murinoglobulin-1	Mug1	down
P10639	11649.63	4	32	1.883	6.6	0.001	Thioredoxin	Txn	down
Q9WVA4	22363.15	4	31	0.641	1.9	0.001	Transgelin-2	Tagln2	down
O35381	28502.23	4	31	0.96	2.6	0.001	Acidic leucine-rich nuclear phosphoprotein 32 family member A	Anp32a	down
Q9CQ43	17355.7	3	30	0.894	2.4	0.002	Deoxyuridine triphosphatase	Dut	down
P34022	23563.7	3	24	0.862	2.4	0.003	Ran-specific GTPase-activating protein	Ranbp1	down
P99024	49620.96	2	23	1.205	3.3	0.005	Tubulin beta-5 chain	Tubb5	down
P56480	56247.46	5	21	1.362	3.9	0.001	ATP synthase subunit beta, mitochondrial	Atp5b	down
Q3U0V1	76709.7	4	20	1.18	3.3	0.001	Far upstream element-binding protein 2	Khsrp	down
O88569	37361.71	3	19	0.584	1.8	0.018	Heterogeneous nuclear ribonucleoproteins A2/B1	Hnrnpa2b1	down
P09411	44503.98	2	19	1.553	4.7	0.007	Phosphoglycerate kinase 1	Pgk1	down
Q64727	116626.3	5	18	0.99	2.7	0.002	Vinculin	Vcl	down
Q91VW3	10452.26	3	18	0.853	2.3	0.008	SH3 domain-binding glutamic acid-rich-like protein 3	Sh3bgrl3	down
P27773	56624.67	3	18	0.635	1.9	0.003	Protein disulfide-isomerase A3	Pdia3	down
P05201	46200.5	4	17	0.547	1.7	0.001	Aspartate aminotransferase, cytoplasmic	Got1	down
Q9JMG1	16340.9	2	17	1.49	4.4	0.005	Endothelial differentiation-related factor 1	Edf1	down
Q62426	11020.53	4	16	0.762	2.1	0.010	Cystatin-B	Cstb	down
P63028	19431.54	2	16	1.959	7.1	0.005	Translationally-controlled tumor protein	Tpt1	down
Q99KC8	87069.41	3	14	1.568	4.8	0.005	von Willebrand factor A domain-containing protein 5A	Vwa5a	down
Q99PT1	23374.8	2	14	0.564	1.8	0.008	Rho GDP-dissociation inhibitor 1	Arhgdia	down
P19157	23576.1	2	14	0.663	1.9	0.008	Glutathione S-transferase P 1	Gstp1	down
P14152	36470.07	2	14	2.03	7.6	0.009	Malate dehydrogenase, cytoplasmic	Mdh1	down
P60335	37455.93	2	14	0.737	2.1	0.007	Poly(rC)-binding protein 1	Pcbp1	down
O35685	38316.28	4	13	0.541	1.7	0.002	Nuclear migration protein nudC	Nudc	down
Q05144	21409.07	2	13	1.987	7.3	0.005	Ras-related C3 botulinum toxin substrate 2	Rac2	down
O08997	7315.65	1	12	0.547	1.7	0.033	Copper transport protein ATOX1	Atox1	down
O08553	62220.58	2	11	0.451	1.6	0.006	Dihydropyrimidinase-related protein 2	Dpysl2	down
P99026	29079.32	1	11	0.527	1.7	0.010	Proteasome subunit beta type-4	Psmb4	down
Q9R0Q7	18691.41	1	10	1.173	3.2	0.010	Prostaglandin E synthase 3	Ptges3	down
P63038	60899.38	1	10	1.179	3.3	0.030	60 kDa heat shock protein, mitochondrial	Hspd1	down
Q91XL1	37389.49	1	10	1.22	3.4	0.010	Leucine-rich HEV glycoprotein	Lrg1	down
Q9CQR2	9117.54	1	10	1.017	2.8	0.010	40S ribosomal protein S21	Rps21	down
Q60865	78102.79	2	9	0.824	2.3	0.007	Caprin-1	Caprin1	down
P07724	68629.7	3	8	0.514	1.7	0.037	Serum albumin	Alb	down
O70591	16505.56	1	7	0.919	2.5	0.010	Prefoldin subunit 2	Pfdn2	down
P63158	24860.15	1	7	0.594	1.8	0.034	High mobility group protein B1	Hmgb1	down
Q6IRU2	28432.42	1	7	3.08	21.8	0.023	Tropomyosin alpha-4 chain	Tpm4	down
P09405	76658.76	1	7	2.151	8.6	0.010	Nucleolin	Ncl	down
Q61823	51651.98	2	6	1.216	3.4	0.010	Programmed cell death protein 4	Pdcd4	down
P23198	20824.33	1	6	0.524	1.7	0.010	Chromobox protein homolog 3	Cbx3	down
Q62418	48651.6	1	5	1.137	3.1	0.010	Drebrin-like protein	Dbnl	down
P30416	51521.93	1	5	0.87	2.4	0.010	Peptidyl-prolyl cis-trans isomerase FKBP4	Fkbp4	down
Q9D1A2	52715.6	1	5	0.535	1.7	0.010	Cytosolic non-specific dipeptidase	Cndp2	down
P32067	47708.95	1	5	1.667	5.3	0.012	Lupus La protein homolog	Ssb	down
Q9DBP5	22133.28	1	4	1.143	3.1	0.010	UMP-CMP kinase	Cmpk1	down
Q9CQQ8	11610.95	1	4	0.467	1.6	0.010	U6 snRNA-associated Sm-like protein LSm7	Lsm7	down
P15105	42074.25	1	4	0.631	1.9	0.010	Glutamine synthetase	Glul	down
Q921M7	36734.64	1	3	1.693	5.4	0.041	Protein FAM49B	Fam49b	down
Q61425	34423.87	1	3	1.282	3.6	0.010	Hydroxyacyl-coenzyme A dehydrogenase, mitochondrial	Hadh	down
Q9CYG7	34238.74	1	3	1.51	4.5	0.019	Mitochondrial import receptor subunit TOM34	Tomm34	down
P10126	50064.09	1	3	3.774	43.6	0.034	Elongation factor 1-alpha 1	Eef1a1	down
Q99LT0	11187.76	1	2	0.474	1.6	0.010	Protein dpy-30 homolog	Dpy30	down
Q9D0T1	14146.54	1	2	0.44	1.6	0.043	NHP2-like protein 1	Nhp2l1	down
Q6P8J7	47425.31	1	2	1.232	3.4	0.010	Creatine kinase S-type, mitochondrial	Ckmt2	down
Q64442	38206.7	1	2	1.349	3.9	0.024	Sorbitol dehydrogenase	Sord	down
P70296	20799.32	1	2	1.648	5.2	0.025	Phosphatidylethanolamine-binding protein 1	Pebp1	down
P60710	41691.72	1	2	1.783	5.9	0.019	Actin, cytoplasmic 1	Actb	down
Q61937	32521.8	1	2	0.536	1.7	0.016	Nucleophosmin	Npm1	down
P70195	29854.24	1	2	0.806	2.2	0.016	Proteasome subunit beta type-7	Psmb7	down
P04247	17040.97	1	2	1.268	3.6	0.026	Myoglobin	Mb	down
Q64339	17868.25	1	2	1.797	6.0	0.010	Ubiquitin-like protein ISG15	Isg15	down

Q9DCW4	27587.97	8	66	-1.18	3.3	0.000	Electron transfer flavoprotein subunit beta	Etfb	up
Q8BH95	31436.2	4	27	-0.729	2.1	0.001	Enoyl-CoA hydratase, mitochondrial	Echs1	up
Q99KI0	85392.01	4	26	-0.697	2.0	0.001	Aconitate hydratase, mitochondrial	Aco2	up
Q8BWT1	41785.43	3	25	-0.615	1.8	0.002	3-ketoacyl-CoA thiolase, mitochondrial	Acaa2	up
Q9WTP6	26433.69	3	23	-0.484	1.6	0.004	Adenylate kinase 2, mitochondrial	Ak2	up
P99028	10409.97	2	23	-0.551	1.7	0.005	Cytochrome b-c1 complex subunit 6, mitochondrial	Uqcrh	up
Q9D2G2	48945.47	3	21	-0.617	1.9	0.003	2-oxoglutarate dehydrogenase complex component E2	Dlst	up
Q91V76	34955.33	2	20	-0.879	2.4	0.005	Ester hydrolase C11orf54 homolog	2 SV=1	up
P51885	38222.6	3	18	-0.543	1.7	0.003	Lumican	Lum	up
Q66JS7	26282.74	2	17	-0.481	1.6	0.005	Igk protein	Igk	up
Q3THE6	20664.39	2	13	-0.787	2.2	0.009	Ferritin	2 SV=1	up
P56391	10046.87	2	12	-0.829	2.3	0.007	Cytochrome c oxidase subunit 6B1	Cox6b1	up
P56375	11852.05	2	12	-0.671	2.0	0.008	Acylphosphatase-2	Acyp2	up
P35505	46128.03	2	10	-0.733	2.1	0.009	Fumarylacetoacetase	Fah	up
Q6LD55	11293.81	1	9	-1.036	2.8	0.010	APOAII	Apoa2	up
Q9CR68	29331.19	1	9	-0.485	1.6	0.010	Cytochrome b-c1 complex subunit Rieske, mitochondrial	Uqcrfs1	up
P20108	28091.4	2	7	-0.723	2.1	0.005	Thioredoxin-dependent peroxide reductase, mitochondrial	Prdx3	up
P37804	22543.37	1	7	-0.651	1.9	0.010	Transgelin	Tagln	up
P09528	21035.25	1	7	-0.629	1.9	0.010	Ferritin heavy chain	Fth1	up
Q8QZT1	44769.33	1	7	-0.642	1.9	0.010	Acetyl-CoA acetyltransferase, mitochondrial	Acat1	up
Q99LC5	34969.49	1	4	-0.598	1.8	0.010	Electron transfer flavoprotein subunit alpha, mitochondrial	Etfa	up
O89020	69315.75	1	4	-0.406	1.5	0.016	Afamin	Afm	up
P00920	28996.49	1	4	-0.431	1.5	0.030	Carbonic anhydrase 2	Ca2	up
P13634	28295.16	1	4	-0.569	1.8	0.010	Carbonic anhydrase 1	Ca1	up
Q99JY0	51335.4	1	3	-1.201	3.3	0.018	Trifunctional enzyme subunit beta, mitochondrial	Hadhb	up
P24472	25529.38	1	3	-0.638	1.9	0.010	Glutathione S-transferase A4	Gsta4	up
P08074	25923.6	1	3	-1.202	3.3	0.010	Carbonyl reductase [NADPH] 2	Cbr2	up
Q9D051	38894.01	1	2	-1.503	4.5	0.010	Pyruvate dehydrogenase E1 component subunit beta, mitochondrial	Pdhb	up
P10493	136432.48	1	2	-0.927	2.5	0.044	Nidogen-1	Nid1	up
Q8BKZ9	53947.22	1	2	-0.99	2.7	0.033	Pyruvate dehydrogenase protein X component, mitochondrial	Pdhx	up
Q8BMF4	67880.67	1	2	-1.22	3.4	0.010	Pyruvate dehydrogenase complex component E2	Dlat	up
O55042	14458.17	1	2	-0.898	2.5	0.010	Alpha-synuclein	Snca	up
P13707	37530.42	1	2	-1.025	2.8	0.031	Glycerol-3-phosphate dehydrogenase [NAD(+)], cytoplasmic	Gpd1	up
P20918	90730.9	1	2	-0.536	1.7	0.039	Plasminogen	Plg	up
