# Supplementary material for: Thymic Microenvironment Is Modified by Malnutrition and Leishmania infantum Infection
Source: Front Cell Infect Microbiol. 2019 Jul 12;9:252. doi: 10.3389/fcimb.2019.00252 (PMC6639785; doi:10.3389/fcimb.2019.00252)
Supplement: Supplementary file 2 [file Table_2.pdf]

Comparison of protein abundance between CPi vs. LPi animals

Description	Cpi	Lpi
Serotransferrin	red	green
Electron transfer flavoprotein subunit beta	red	green
Hemopexin	red	green
Fumarate hydratase, mitochondrial	red	green
Cytochrome c oxidase subunit 6B1	red	green
Enoyl-CoA hydratase, mitochondrial	red	green
3-ketoacyl-CoA thiolase, mitochondrial	red	green
Vitamin D-binding protein	red	green
Malate dehydrogenase, cytoplasmic	red	green
Fumarylacetoacetase	red	green
Ferritin	red	green
Ester hydrolase C11orf54 homolog	red	green
Acylphosphatase-2	red	green
Malate dehydrogenase, mitochondrial	red	green
2-oxoglutarate dehydrogenase complex component E2	red	green
Electron transfer flavoprotein subunit alpha, mitochondrial	red	green
Creatine kinase S-type, mitochondrial	red	green
Serine protease inhibitor A3K	red	red
Carbonic anhydrase 3	red	red
Glutathione S-transferase P 1	red	red
Triosephosphate isomerase	red	red
Heterogeneous nuclear ribonucleoproteins A2/B1	red	red
ATP synthase subunit beta, mitochondrial	red	red
Acidic leucine-rich nuclear phosphoprotein 32 family member A	red	red
Phosphoglycerate kinase 1	red	red
Protein disulfide-isomerase A3	red	red
60 kDa heat shock protein, mitochondrial	red	red
14-3-3 protein zeta/delta	red	red
Nucleoside diphosphate kinase	red	red
Poly(rC)-binding protein 1	red	red
Transitional endoplasmic reticulum ATPase	red	red
Transcription elongation factor B polypeptide 2	red	red
Annexin A5	red	red
Dihydropyrimidinase-related protein 2	red	red
Aspartate aminotransferase, cytoplasmic	red	grey
Cystatin-B	red	grey
Serum albumin	red	grey
Vinculin	red	grey
Thioredoxin-dependent peroxide reductase, mitochondrial	red	grey
Glutathione S-transferase A4	red	grey
Cytochrome c oxidase subunit 5A, mitochondrial	red	grey
Lumican	red	grey
Glutamine synthetase	red	grey
Protein dpy-30 homolog	red	grey
Non-specific lipid-transfer protein	red	grey
Nucleophosmin	red	grey
Proteasome subunit beta type-7	red	grey
Thioredoxin domain-containing protein 17	green	red
Cytosolic non-specific dipeptidase	green	red
Actin, cytoplasmic 1	green	red
Microtubule-associated protein 4	green	grey
Programmed cell death protein 4	green	grey
Elongation factor 1-alpha 1	green	grey
Eukaryotic translation initiation factor 4E	green	grey
Prothymosin alpha	green	grey
Myristoylated alanine-rich C-kinase substrate	grey	red
Keratin, type II cytoskeletal 8	grey	red
Keratin, type I cytoskeletal 18	grey	red
Galectin-1	grey	red
60S acidic ribosomal protein P2	grey	red
40S ribosomal protein S12	grey	red
Rho GDP-dissociation inhibitor 2	grey	red
Murinoglobulin-1	grey	red
Thioredoxin	grey	red
Transgelin-2	grey	red
Ran-specific GTPase-activating protein	grey	red
Far upstream element-binding protein 2	grey	red
SH3 domain-binding glutamic acid-rich-like protein 3	grey	red
Endothelial differentiation-related factor 1	grey	red
Translationally-controlled tumor protein	grey	red
von Willebrand factor A domain-containing protein 5A	grey	red
Rho GDP-dissociation inhibitor 1	grey	red
Nuclear migration protein nudC	grey	red
Ras-related C3 botulinum toxin substrate 2	grey	red
Copper transport protein ATOX1	grey	red
Proteasome subunit beta type-4	grey	red
40S ribosomal protein S21	grey	red
High mobility group protein B1	grey	red
Tropomyosin alpha-4 chain	grey	red
Nucleolin	grey	red

Number of proteins that changed in LPi when compared to CPi

34	green	upregulated
35	red	downregulated
18	grey	proteins that were altered in CPi animals (up-green or down-red) but were not altered in LPi

Drebrin-like protein		
Peptidyl-prolyl cis-trans isomerase FKBP4		
Lupus La protein homolog		
UMP-CMP kinase		
U6 snRNA-associated Sm-like protein LSm7		
NHP2-like protein 1		
Carbonic anhydrase 2		
Peroxiredoxin-2		
Malate dehydrogenase, cytoplasmic		
Leucine-rich HEV glycoprotein		
Hydroxyacyl-coenzyme A dehydrogenase, mitochondrial		
Aconitate hydratase, mitochondrial		
Adenylate kinase 2, mitochondrial		
Cytochrome b-c1 complex subunit 6, mitochondrial		
APOAII		
Cytochrome b-c1 complex subunit Rieske, mitochondrial		
Transgelin		
Ferritin heavy chain		
Trifunctional enzyme subunit beta, mitochondrial		
Nidogen-1		
Pyruvate dehydrogenase protein X component, mitochondrial		
Pyruvate dehydrogenase complex component E2		
Alpha-synuclein		
Plasminogen		

Comparison of protein abundance between LP vs. LPi animals

Description	LP	LPi
Serotransferrin	red	green
Hemopexin	red	green
Peroxiredoxin-2	red	green
Malate dehydrogenase, cytoplasmic	red	green
Leucine-rich HEV glycoprotein	red	green
Hydroxyacyl-coenzyme A dehydrogenase, mitochondrial	red	green
Creatine kinase S-type, mitochondrial	red	green
Serine protease inhibitor A3K	red	red
Annexin A5	red	red
Carbonic anhydrase 3	red	red
Transitional endoplasmic reticulum ATPase	red	red
Myristoylated alanine-rich C-kinase substrate	red	red
Keratin, type II cytoskeletal 8	red	red
Keratin, type I cytoskeletal 18	red	red
14-3-3 protein zeta/delta	red	red
Galectin-1	red	red
60S acidic ribosomal protein P2	red	red
40S ribosomal protein S12	red	red
Rho GDP-dissociation inhibitor 2	red	red
Murinoglobulin-1	red	red
Thioredoxin	red	red
Transgelin-2	red	red
Acidic leucine-rich nuclear phosphoprotein 32 family member A	red	red
Ran-specific GTPase-activating protein	red	red
ATP synthase subunit beta, mitochondrial	red	red
Far upstream element-binding protein 2	red	red
Heterogeneous nuclear ribonucleoproteins A2/B1	red	red
Phosphoglycerate kinase 1	red	red
SH3 domain-binding glutamic acid-rich-like protein 3	red	red
Protein disulfide-isomerase A3	red	red
Endothelial differentiation-related factor 1	red	red
Translationally-controlled tumor protein	red	red
von Willebrand factor A domain-containing protein 5A	red	red
Rho GDP-dissociation inhibitor 1	red	red
Glutathione S-transferase P 1	red	red
Poly(rC)-binding protein 1	red	red
Nuclear migration protein nudC	red	red
Ras-related C3 botulinum toxin substrate 2	red	red
Copper transport protein ATOX1	red	red
Dihydropyrimidinase-related protein 2	red	red
Proteasome subunit beta type-4	red	red
60 kDa heat shock protein, mitochondrial	red	red
40S ribosomal protein S21	red	red
High mobility group protein B1	red	red
Tropomyosin alpha-4 chain	red	red
Nucleolin	red	red
Drebrin-like protein	red	red
Peptidyl-prolyl cis-trans isomerase FKBP4	red	red
Lupus La protein homolog	red	red
UMP-CMP kinase	red	red
U6 snRNA-associated Sm-like protein LSm7	red	red
NHP2-like protein 1	red	red
Actin, cytoplasmic 1	red	red
Nucleoside diphosphate kinase	red	grey
Pyruvate kinase PKM	red	grey
Deoxyuridine triphosphatase	red	grey
Tubulin beta-5 chain	red	grey
Vinculin	red	grey
Aspartate aminotransferase, cytoplasmic	red	grey
Cystatin-B	red	grey
Prostaglandin E synthase 3	red	grey
Caprin-1	red	grey
Serum albumin	red	grey
Prefoldin subunit 2	red	grey
Programmed cell death protein 4	red	grey
Chromobox protein homolog 3	red	grey
Cytosolic non-specific dipeptidase	red	grey
Glutamine synthetase	red	grey
Protein FAM49B	red	grey
Mitochondrial import receptor subunit TOM34	red	grey
Elongation factor 1-alpha 1	red	grey
Protein dpy-30 homolog	red	grey
Sorbitol dehydrogenase	red	grey
Phosphatidylethanolamine-binding protein 1	red	grey
Nucleophosmin	red	grey
Proteasome subunit beta type-7	red	grey
Myoglobin	red	grey
Ubiquitin-like protein ISG15	red	grey
Carbonic anhydrase 2	green	red
Lumican	green	grey
Igk protein	green	grey
Thioredoxin-dependent peroxide reductase, mitochondrial	green	grey
Acetyl-CoA acetyltransferase, mitochondrial	green	grey
Afamin	green	grey
Carbonic anhydrase 1	green	grey
Glutathione S-transferase A4	green	grey

Number of proteins that changed in LPi when compared to LP

7	green	upregulated
1	red	downregulated
35	grey	proteins that were altered in LP animals (up-green or down-red) but were not altered in LPi

Description	Cpi	LP	Lpi
Electron transfer flavoprotein subunit beta	downregulated	upregulated	upregulated
Enoyl-CoA hydratase, mitochondrial	downregulated	upregulated	upregulated
3-ketoacyl-CoA thiolase, mitochondrial	downregulated	upregulated	upregulated
2-oxoglutarate dehydrogenase complex component E2	downregulated	upregulated	upregulated
Ester hydrolase C11orf54 homolog	downregulated	upregulated	upregulated
Ferritin	downregulated	upregulated	upregulated
Cytochrome c oxidase subunit 6B1	downregulated	upregulated	upregulated
Acylphosphatase-2	downregulated	upregulated	upregulated
Fumarylacetoacetase	downregulated	upregulated	upregulated
Electron transfer flavoprotein subunit alpha, mitochondrial	downregulated	upregulated	upregulated
Lumican	downregulated	upregulated	unchanged
Thioredoxin-dependent peroxide reductase, mitochondrial	downregulated	upregulated	unchanged
Glutathione S-transferase A4	downregulated	upregulated	unchanged
Fumarate hydratase, mitochondrial	downregulated	unchanged	upregulated
Vitamin D-binding protein	downregulated	unchanged	upregulated
Malate dehydrogenase, cytoplasmic	downregulated	unchanged	upregulated
Malate dehydrogenase, mitochondrial	downregulated	unchanged	upregulated
Serotransferrin	downregulated	downregulated	upregulated
Hemopexin	downregulated	downregulated	upregulated
Creatine kinase S-type, mitochondrial	downregulated	downregulated	upregulated
Serine protease inhibitor A3K	downregulated	downregulated	downregulated
Annexin A5	downregulated	downregulated	downregulated
Carbonic anhydrase 3	downregulated	downregulated	downregulated
Transitional endoplasmic reticulum ATPase	downregulated	downregulated	downregulated
14-3-3 protein zeta/delta	downregulated	downregulated	downregulated
Acidic leucine-rich nuclear phosphoprotein 32 family member A	downregulated	downregulated	downregulated
ATP synthase subunit beta, mitochondrial	downregulated	downregulated	downregulated
Heterogeneous nuclear ribonucleoproteins A2/B1	downregulated	downregulated	downregulated
Phosphoglycerate kinase 1	downregulated	downregulated	downregulated
Protein disulfide-isomerase A3	downregulated	downregulated	downregulated
Glutathione S-transferase P 1	downregulated	downregulated	downregulated
Poly(rC)-binding protein 1	downregulated	downregulated	downregulated
Dihydropyrimidinase-related protein 2	downregulated	downregulated	downregulated
60 kDa heat shock protein, mitochondrial	downregulated	downregulated	downregulated
Nucleoside diphosphate kinase	downregulated	downregulated	unchanged
Vinculin	downregulated	downregulated	unchanged
Aspartate aminotransferase, cytoplasmic	downregulated	downregulated	unchanged
Cystatin-B	downregulated	downregulated	unchanged
Serum albumin	downregulated	downregulated	unchanged
Glutamine synthetase	downregulated	downregulated	unchanged
Protein dpy-30 homolog	downregulated	downregulated	unchanged
Nucleophosmin	downregulated	downregulated	unchanged
Proteasome subunit beta type-7	downregulated	downregulated	unchanged
Triosephosphate isomerase	downregulated	unchanged	downregulated
Transcription elongation factor B polypeptide 2	downregulated	unchanged	downregulated
Cytochrome c oxidase subunit 5A, mitochondrial	downregulated	unchanged	unchanged
Non-specific lipid-transfer protein	downregulated	unchanged	unchanged
Myristoylated alanine-rich C-kinase substrate	unchanged	downregulated	downregulated
Keratin, type II cytoskeletal 8	unchanged	downregulated	downregulated
Keratin, type I cytoskeletal 18	unchanged	downregulated	downregulated
Galectin-1	unchanged	downregulated	downregulated
60S acidic ribosomal protein P2	unchanged	downregulated	downregulated
40S ribosomal protein S12	unchanged	downregulated	downregulated
Rho GDP-dissociation inhibitor 2	unchanged	downregulated	downregulated
Murinoglobulin-1	unchanged	downregulated	downregulated
Thioredoxin	unchanged	downregulated	downregulated

upregulated (green)
downregulated (red)
unchanged (grey)

Transgelin-2			
Ran-specific GTPase-activating protein			
Far upstream element-binding protein 2			
SH3 domain-binding glutamic acid-rich-like protein 3			
Endothelial differentiation-related factor 1			
Translationally-controlled tumor protein			
von Willebrand factor A domain-containing protein 5A			
Rho GDP-dissociation inhibitor 1			
Nuclear migration protein nudC			
Ras-related C3 botulinum toxin substrate 2			
Copper transport protein ATOX1			
Proteasome subunit beta type-4			
40S ribosomal protein S21			
High mobility group protein B1			
Tropomyosin alpha-4 chain			
Nucleolin			
Drebrin-like protein			
Peptidyl-prolyl cis-trans isomerase FKBP4			
Lupus La protein homolog			
UMP-CMP kinase			
U6 snRNA-associated Sm-like protein LSm7			
NHP2-like protein 1			
Deoxyuridine triphosphatase			
Tubulin beta-5 chain			
Prostaglandin E synthase 3			
Caprin-1			
Prefoldin subunit 2			
Chromobox protein homolog 3			
Protein FAM49B			
Mitochondrial import receptor subunit TOM34			
Sorbitol dehydrogenase			
Phosphatidylethanolamine-binding protein 1			
Myoglobin			
Pyruvate kinase PKM			
Ubiquitin-like protein ISG15			
Programmed cell death protein 4			
Cytosolic non-specific dipeptidase			
Elongation factor 1-alpha 1			
Peroxiredoxin-2			
Malate dehydrogenase, cytoplasmic			
Leucine-rich HEV glycoprotein			
Hydroxyacyl-coenzyme A dehydrogenase, mitochondrial			
Actin, cytoplasmic 1			
Thioredoxin domain-containing protein 17			
Carbonic anhydrase 2			
Acetyl-CoA acetyltransferase, mitochondrial			
Afamin			
Carbonic anhydrase 1			
Carbonyl reductase [NADPH] 2			
Pyruvate dehydrogenase E1 component subunit beta, mitochondrial			
Igk protein			
Glycerol-3-phosphate dehydrogenase [NAD(+)], cytoplasmic			
Microtubule-associated protein 4			
Eukaryotic translation initiation factor 4E			
Prothymosin alpha			
Aconitate hydratase, mitochondrial			
Adenylate kinase 2, mitochondrial			
Cytochrome b-c1 complex subunit 6, mitochondrial			

APOAII			
Cytochrome b-c1 complex subunit Rieske, mitochondrial			
Transgelin			
Ferritin heavy chain			
Plasminogen			
Nidogen-1			
Pyruvate dehydrogenase protein X component, mitochondrial			
Pyruvate dehydrogenase complex component E2			
Alpha-synuclein			
Trifunctional enzyme subunit beta, mitochondrial			
