# Supplementary material for: Thymic Microenvironment Is Modified by Malnutrition and Leishmania infantum Infection
Source: Front Cell Infect Microbiol. 2019 Jul 12;9:252. doi: 10.3389/fcimb.2019.00252 (PMC6639785; doi:10.3389/fcimb.2019.00252)
Supplement: Supplementary file 3 [file Data_Sheet_1.pdf]

Supplementary Material

Tables:

Supplementary Table 1 - sheet 1. Identified proteins in thymic IF. **Sa** Non-classic secretion. **Sp**. Signal peptide of secretion; **– sheet 2.** Single peptide identified proteins in thymic IF. **Sa** Non-classic secretion. **Sp**. Signal peptide of secretion; **– sheet 3.** Differentially abundant proteins in the IF of the thymus of well-nourished mice infected with *L. infantum* BALB/c mice (CPi); **– sheet 4.** Differentially abundant proteins in the IF of the thymus of protein malnourished BALB/c mice (LP).

Supplementary Table 2 - sheet 1. Comparison of protein abundance between CPi vs. LPi animals. **– sheet 2.** Comparison of protein abundance between LP vs. LPi animals; **– sheet 3.** Graphical comparison of protein abundance among CPi, LP and LPi animals.

Figures:

Supplementary Figure 1. Protein quantification in thymic interstitial fluid using T-test. Peptides are mapped as circles on the plot according to its –Log(p-value) (x-axis) and Log (Fold change) (y-axis). Green circles indicated peptides having a higher abundance in condition CP, and red circles indicate those with a higher abundance in condition (A) LP, (B) CPi and (C) LPi. The gray translucent circles indicated peptides that did not pass the criteria that satisfy neither the variable fold-change cutoff nor the FDR cutoff $\alpha = 0.05$.

Supplementary Figure 2. Functional interaction networks of differentially abundant proteins identified in the thymic IF of CPi and LP mice grouped by biological process. Proteins with differential abundance between the CP group and the (A) CPi or (B) LP group in the thymic IF were grouped according to the biological process enriched with $p < 0.05$. The proteins represented in the central circles did not present clusters with significant enrichment in the biological process. Network was built using the IIS (Integrated interactome system) platform and viewed in Cytoscape software version 2.8.3.

Supplementary Figure 3. Lymphocyte subpopulations in the thymus of malnourished BALB/c mice infected with *L. infantum*. Total CD4+, CD8+ and DP subpopulations were analyzed by flow cytometry. Each T cell subpopulation is expressed relative to the total number of cells ± SEM. Statistical differences due to diet (**a**, $p < 0.0001$), infection (**b**, $p < 0.05$) or an interaction between diet and infection (**c**, $p < 0.05$) were determined by two-way ANOVA with Bonferroni post-hoc test. CP: animals fed 14% protein diet; LP: animals fed 4% protein diet, CPi: animals fed 14% protein diet and infected; LPi: animals fed 4% protein diet and infected.

Supplementary Figure 4. Gate strategy. To identify the population of interest we performed the following gate strategy: by first gating on alive (SSC versus FSC) and singlets (FSC-H versus FSC-A) cells, we defined the CD4 T cells as CD4+CD8-, CD8 T

cells as CD4-CD8+ and double positive (DP) T cells as CD4+CD8+ T cells. A Fluorescence Minus One (FMO) Control was used to identify and gate the Ki67+ cells.
