# Supplementary figures and images for: Thymic Microenvironment Is Modified by Malnutrition and Leishmania infantum Infection
Source: Front Cell Infect Microbiol. 2019 Jul 12;9:252. doi: 10.3389/fcimb.2019.00252 (PMC6639785; doi:10.3389/fcimb.2019.00252)

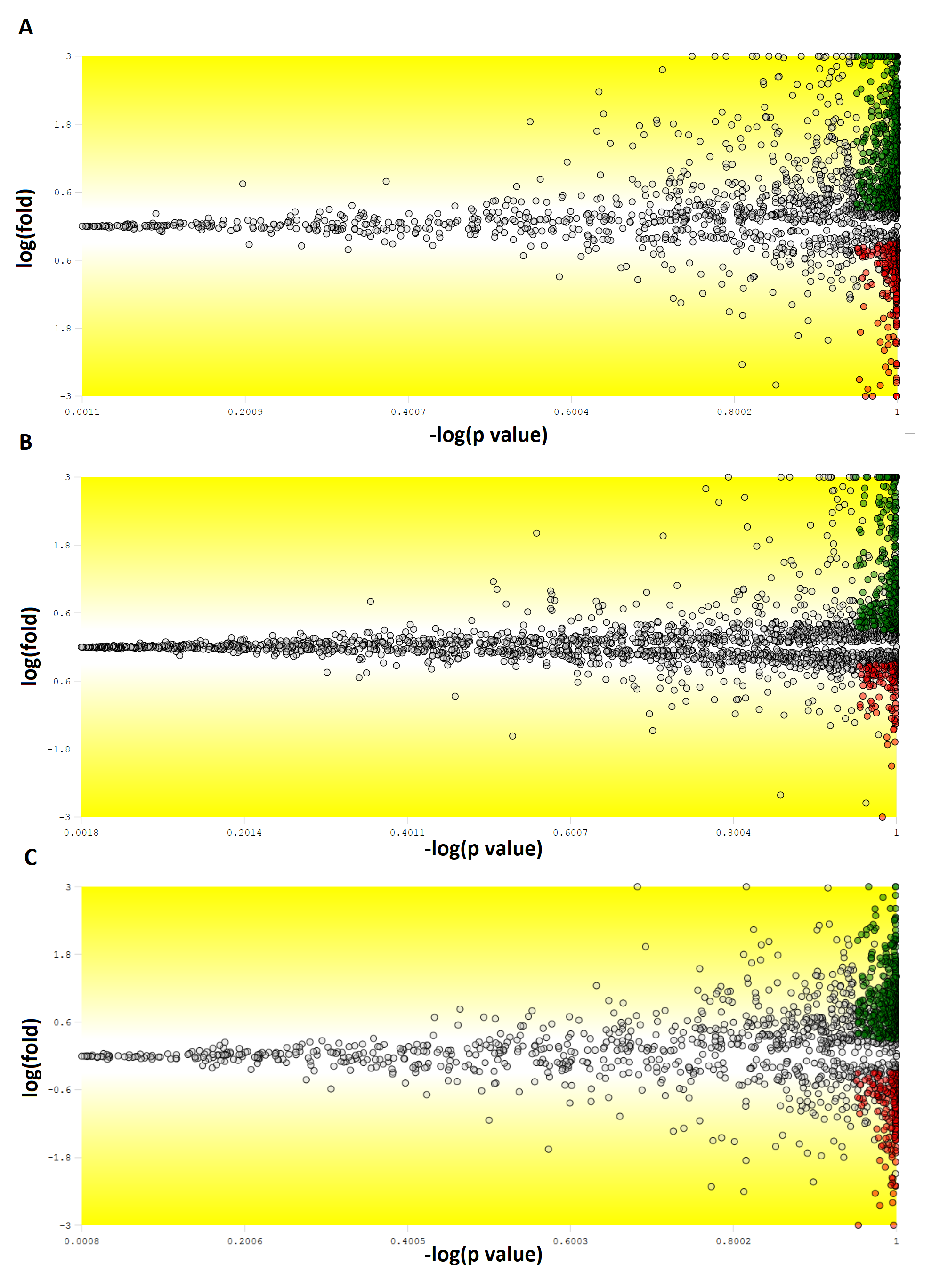

Supplement: Supplementary file 4 [file Image_1.TIF]

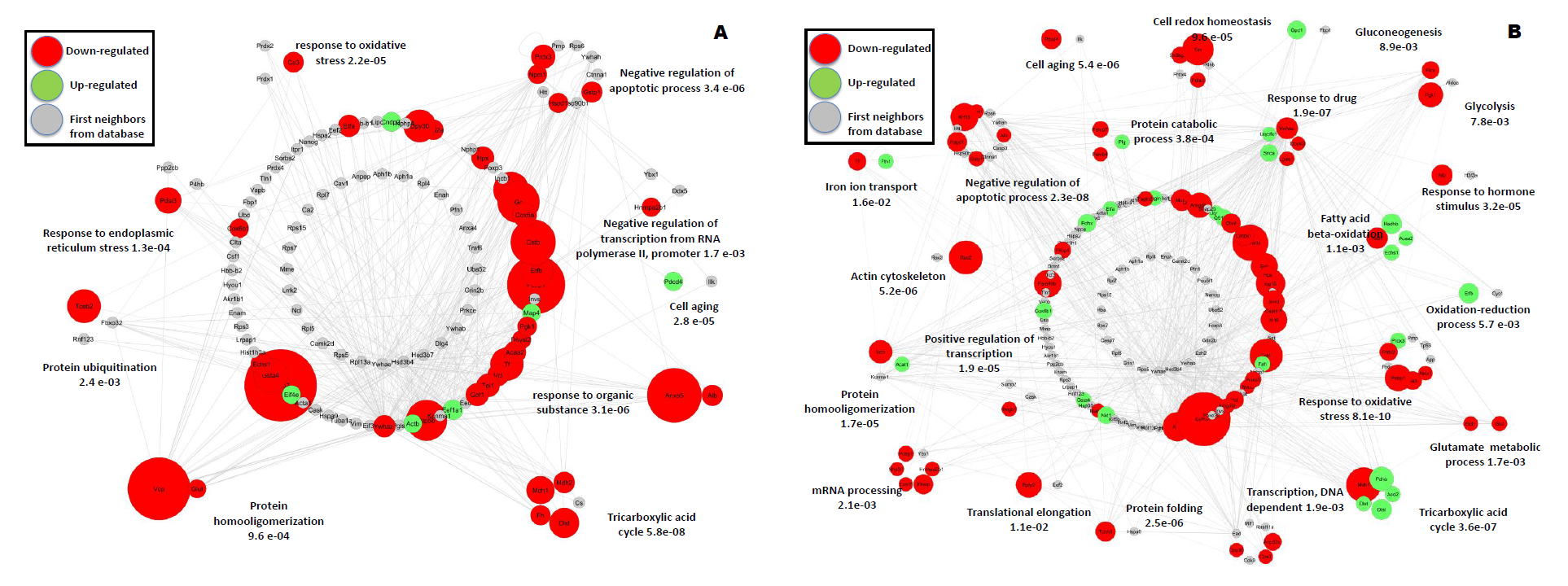

Supplement: Supplementary file 5 [file Image_2.tif]

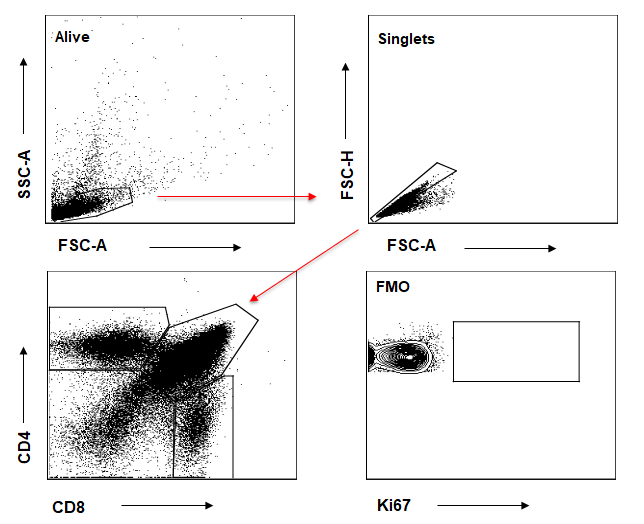

Supplement: Supplementary file 7 [file Image_4.tif]
